# Effects of Presentation Form and Presentation Timing in AR-HUD Takeover Displays on Driver Visual Attention: An Eye-Tracking Study

**DOI:** 10.3390/jemr19040092

**Published:** 2026-08-20

**Authors:** Kexin Chen, Junfeng Li, Mo Chen

**Affiliations:** College of Art and Design, Nanjing Tech University, Nanjing 211816, China; ckx7228nice@163.com (K.C.); thisisjunfengli@njtech.edu.cn (J.L.)

**Keywords:** eye-tracking, AR-HUD, takeover request, presentation timing, presentation form, visual attention

## Abstract

Level 3 automated driving necessitates rapid driver reengagement following a takeover request, making human–machine interface design critical for safety. As a sensor-based in-vehicle interface, the augmented reality head-up display (AR-HUD) integrates real-time environmental sensing with visual information delivery, yet how different information presentation strategies influence driver visual attention during takeover remains underexplored. We examined the effects of presentation form (static/dynamic) and presentation timing (concurrent/progressive) using a 2 × 2 within-subject design. In the experiment, twenty-one licensed drivers viewed prerecorded automated driving takeover scenarios. Eye tracking measured mean fixation duration, mean saccade amplitude, time to first fixation, and fixation count, while ratings assessed usability, acceptance, and intention comprehension. Progressive presentation significantly reduced all eye-tracking measures and improved perceived usability ratings compared with concurrent presentation. This pattern indicates more concentrated and orderly gaze allocation and is consistent with lower visual-search and information-integration demands. However, the lower fixation count may partly reflect reduced information exposure. Static presentation reduced mean fixation duration but showed no consistent overall advantage on the remaining measures. Significant form-by-timing interactions showed that progressive presentation reduced saccade amplitude and time to first fixation and improved intention comprehension under static, but not dynamic, presentation. Among the four combinations, static-progressive presentation yielded the most favorable overall pattern. Overall, presentation timing affected more measured outcomes than presentation form. These findings provide preliminary evidence that presentation form and presentation timing shape gaze behavior and subjective evaluations of AR-HUD takeover displays.

## 1. Introduction

With the commercialization of Society of Automotive Engineers (SAE) Level 3 (L3) conditional automated driving, ensuring safety during the transition of vehicle control between the automated system and the human driver has become a critical issue for both academia and industry. Unlike lower levels of driving automation, L3 automated driving allows drivers to disengage from continuous monitoring of the driving environment and perform non-driving-related tasks (NDRTs). However, when a takeover request (TOR) is issued, drivers are required to regain control of the vehicle within a limited takeover time budget (TTB) [1,2]. Previous studies have demonstrated that the transition from an automation supervisor to an active driver is not instantaneous but involves multiple sequential cognitive processes, including attentional reallocation, recovery of situation awareness (SA), and decision-making [3,4]. According to Endsley’s three-level SA model [3], drivers must perceive environmental elements, comprehend the current situation, and project future system states before executing appropriate control actions. Delays or deficiencies at any stage may impair takeover quality. Existing evidence further indicates that drivers exhibit significantly lower SA during automated driving than during manual driving [5,6], while shorter TTBs further increase the difficulty of cognitive recovery [7,8]. Consequently, improving the efficiency of information acquisition during TOR has become a major objective in the design of human–machine interfaces (HMIs) for automated vehicles.

As an emerging HMI technology for intelligent cockpits, Augmented Reality Head-Up Displays (AR-HUDs) integrate multi-source environmental perception with real-time information visualization. By fusing data acquired from onboard sensing systems—including cameras, millimeter-wave radar, LiDAR, and high-definition maps—AR-HUDs superimpose virtual driving cues directly onto the driver’s real-world field of view, enabling intuitive visualization of environmental perception results. Compared with conventional HUDs or center console displays, AR-HUDs reduce gaze deviation and facilitate faster information acquisition, making them a promising platform for presenting TOR-related information [9,10]. Previous studies have reported that AR-HUDs can improve driving performance, vehicle stability, and situation awareness [9,11]. Nevertheless, during the limited takeover window, excessive virtual overlays may compete with real traffic information, resulting in visual distraction, attentional interference, and occlusion of safety-critical objects [12]. For example, Smith et al. [13] observed that some drivers continued focusing on AR graphics instead of shifting attention to actual hazards during emergency situations. Huang et al. [14] further demonstrated that the organization of TOR interface information directly affects takeover quality and driving safety. These findings suggest that effective information presentation strategies for AR-HUDs remain an important research challenge.

Within the broader literature on TOR interfaces, recent studies have increasingly combined visual, auditory, and tactile cues to create multimodal TORs. Auditory and tactile cues can complement visual warnings and may accelerate attentional reorientation and information processing. However, their benefits have not been consistent across response time, vehicle control, and subjective evaluations [15,16,17]. This inconsistency indicates that their effectiveness depends on the functions of individual cues and how these cues interact. Existing research has focused primarily on the selection and combination of sensory modalities, whereas comparatively little attention has been paid to presentation timing and presentation form within the visual modality.

Existing studies have primarily investigated two design dimensions of AR-HUD information presentation, namely presentation form and presentation timing. Regarding presentation form, most studies have compared static visual information with dynamic visual encoding. Dynamic visual cues, such as flashing, motion, and looming effects, possess higher visual salience and can rapidly capture attention [18]. However, multimedia learning research suggests that continuous animations do not necessarily outperform static representations, as transient visual changes may increase cognitive load and distract users from task-relevant information [19]. Similar findings have been reported in AR-HUD applications. Zhai et al. [20] found that flashing cues improved drivers’ hazard-time estimation, although different flashing patterns produced no significant differences. Wu et al. [21] demonstrated that dynamically tracking warning icons enhanced driving performance by maintaining spatial consistency between warning information and hazardous objects. In contrast, Solombrino et al. [22] argued that dynamic visualizations reduce cognitive workload only when their temporal evolution is predictable; otherwise, dynamic information may become counterproductive. Therefore, whether dynamic presentation is superior to static presentation largely depends on task characteristics and information organization [23].

On the other hand, the timing of information presentation also affects the driver’s information processing efficiency. During the TOR process, information such as risk warnings, explanations of causes, and action guidelines can be presented simultaneously or unfolded gradually in accordance with the driver’s cognitive process. Cognitive Load Theory (CLT) posits that working memory capacity is limited, and that appropriately controlling the pace of information presentation helps reduce cognitive load [24,25]. However, current research findings on progressive information presentation are inconsistent. Anik and Bunt [26] found that progressive disclosure improves users’ subjective experience but does not significantly reduce objective cognitive load. In AR-HUD takeover scenarios, Zhu et al. [27] confirmed that different timing of prompts significantly affects drivers’ situation awareness and cognitive load, suggesting that there is an optimal time window for information presentation. Nevertheless, existing studies have primarily focused on the lead time of prompts, with few systematically comparing concurrent and progressive presentation as independent variables; the underlying design principles of such approaches still require further validation.

It is worth noting that the form of information presentation and the timing of presentation are inherently coupled. Dynamic visual elements (such as flashing, gradients, and flowing animations) rely on temporal changes, and their ability to guide attention may be modulated by the rhythm of information presentation. Therefore, examining a single design factor alone is insufficient to reveal the mechanisms underlying AR-HUD information organization. Few existing studies have used orthogonal experimental designs to analyze the interaction between presentation form and presentation timing, and it remains unclear whether dynamic presentation can produce a synergistic effect with progressive presentation, thereby further enhancing drivers’ information acquisition efficiency and visual attention allocation.

With recent advances in optical sensing technology, eye tracking has become an important non-invasive method for examining drivers’ gaze behavior. Compared with retrospective questionnaires, eye tracking continuously records gaze position with millisecond-level temporal resolution, providing objective measures of visual attention allocation during driving [28]. Eye-tracking measures, including fixation duration, time to first fixation, and saccade amplitude, characterize fixation and visual-search behavior [27,29,30]. Combining eye-tracking measures with subjective ratings therefore enables a broader assessment of gaze behavior and interface evaluations across AR-HUD presentation conditions.

To address the above research gaps, this study employed a 2 × 2 within-subject experimental design with two levels of presentation form (static vs. dynamic) and two levels of presentation timing (concurrent vs. progressive). Eye-tracking measures and subjective evaluation measures were used to examine their effects on gaze behavior and subjective evaluations.

Specifically, this study addresses the following research questions:

RQ1: How do presentation form (static vs. dynamic) and presentation timing (concurrent vs. progressive) individually influence drivers’ gaze behavior and subjective evaluations during takeover?

RQ2: Is there an interaction between presentation form and presentation timing in their effects on gaze behavior and subjective evaluations?

RQ3: Are objective eye-tracking measures consistent with subjective evaluation results when assessing different AR-HUD information presentation strategies?

The main contribution of this study is the integration of presentation form and presentation timing within a unified orthogonal experimental framework, enabling comparison of their main and interaction effects on eye-tracking measures and subjective evaluations. The findings provide comparative empirical evidence for understanding the differences among various visual information organization strategies and offer preliminary design guidance for selecting presentation strategies in AR-HUD takeover interfaces.

## 2. Materials and Methods

### 2.1. Participants

Prior to conducting the formal experiment, this study used G*Power 3.1.9.7 software to perform a power analysis to determine the required sample size. Based on the study’s 2 (presentation form) × 2 (presentation timing) within-subjects design, the calculations indicated that at least 19 participants were required under conditions of a medium effect size (f = 0.25), a significance level of α = 0.05, and a statistical power of 0.80. Ultimately, 24 participants were recruited. During eye-tracking data preprocessing, three participants were excluded because their tracking ratios were below 75%. The final sample therefore comprised 21 participants. The participants included 11 men (52.4%) and 10 women (47.6%), ranging in age from 20 to 30 (M = 24.62, SD = 2.44). All participants held a valid driver’s license issued in the People’s Republic of China, had driven within the previous year, and reported an average driving experience of 3.60 years (*SD* = 2.41).

Regarding familiarity with automated driving and advanced driver assistance systems (ADASs), 9.5% of participants reported no prior knowledge, 52.4% were aware of the technology but had never used it, 23.8% had occasional experience, and 14.3% reported frequent use. In addition, 61.9% expressed positive expectations toward automated driving technology.

All participants had normal or corrected-to-normal vision (visual acuity ≥ 5.0) and reported no color vision deficiency. Written informed consent was obtained from all participants before the experiment. The study was conducted in accordance with the relevant ethical standards.

### 2.2. Apparatus

Eye-tracking data were collected using a 7invensun aSee Pro desktop eye tracker (7invensun, Beijing, China). The system employs infrared illumination together with corneal-reflection and dark-pupil tracking techniques to estimate gaze position in real time. It provides a sampling rate of 250 Hz with a spatial accuracy of 0.5°, enabling reliable and non-invasive eye-tracking measurements.

**Figure 1:**
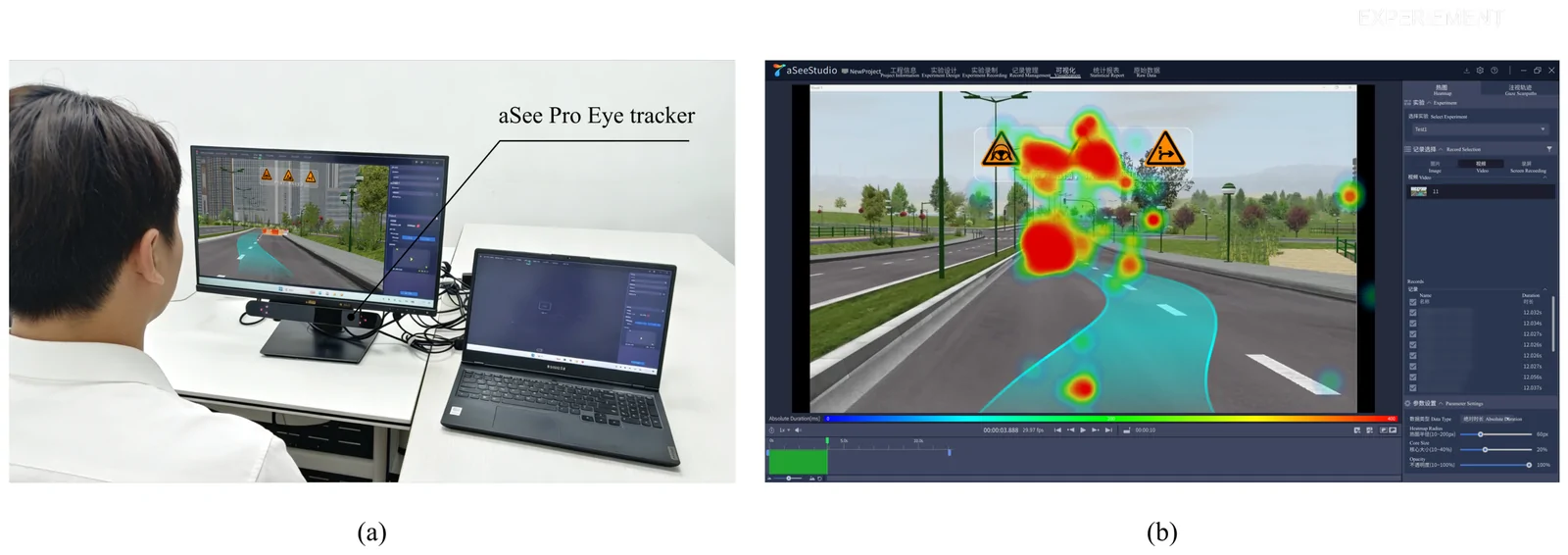
Experimental setup and data acquisition system: (**a**) experimental setup; (**b**) software interface.

The experimental setup is illustrated in Figure 1a. The eye tracker was positioned below the display, with a viewing distance maintained at 65–70 cm. Participants were instructed to minimize head movements throughout the experiment to ensure stable eye-tracking performance. The experiment was conducted on a Lenovo Legion Y7000P laptop (Lenovo Group Ltd., Beijing, China). Autonomous driving takeover scenario videos were presented on a 24-inch ViewSonic VG2481-4K monitor (ViewSonic Corporation, Brea, CA, USA; 3840 × 2160 pixels, 60 Hz). The 1920 × 1080 stimulus videos were displayed full-screen and proportionally scaled to the 3840 × 2160 monitor resolution.

Data acquisition and post-processing were performed using the aSee Studio software platform (version 0.3.40.4) (see Figure 1b). Eye movement events were classified using the software’s built-in I-VT (Identification by Velocity Threshold) algorithm. The velocity threshold was set to 3.0 px/ms, and the minimum fixation duration was set to 60 ms. Based on this velocity-based classification, gaze samples with velocities below the threshold and lasting longer than the minimum duration were identified as fixations, whereas gaze samples exceeding the velocity threshold were classified as saccades.

### 2.3. Experimental Design and Stimuli

This study employed a 2 (presentation form: static vs. dynamic) × 2 (presentation timing: concurrent vs. progressive) within-subject experimental design, resulting in four experimental conditions. To minimize expectancy effects, two representative urban automated driving takeover scenarios were developed for each condition: vehicle breakdown ahead and road construction ahead, yielding a total of eight stimulus videos. Because the study focused on the effects of AR-HUD presentation strategies rather than differences between takeover-event types, the two driving scenarios were treated as repeated trials within each condition. For each participant and experimental condition, eye-tracking measures and questionnaire scores were separately averaged across the two scenarios, yielding one condition-level value for each outcome measure for statistical analysis.

To minimize potential confounding effects, all scenarios were carefully controlled for traffic density, road complexity, and takeover urgency. Specifically, the takeover time budget (TTB) was fixed at 7 s [31,32], defined as the interval between the issuance of the takeover request (TOR) and the potential collision point. Once presented, each information element remained visible until the end of the 7 s takeover period.

#### 2.3.1. TOR Interface Design

The takeover request (TOR) interface was developed based on Endsley’s Situation Awareness (SA) theory [3] and the Situation Awareness-Based Agent Transparency (SAT) model proposed by Chen et al. [33]. According to the SAT model, interface transparency is organized into three hierarchical information levels, What (system status or action), Why (decision rationale), and How (recommended action), corresponding to the three levels of SA, namely perception, comprehension, and projection.

Following this framework, the TOR interface consisted of three consecutive information modules representing the What–Why–How hierarchy. The What module notified drivers that a takeover was required, the Why module explained the environmental cause of the takeover request, and the How module provided the recommended driving maneuver to facilitate rapid understanding of the traffic situation and subsequent action planning. These three modules together form the complete TOR interface, which is designed to help drivers quickly obtain key environmental information within a limited takeover window and assist them in understanding the requirements of the takeover task.

**Figure 2:**
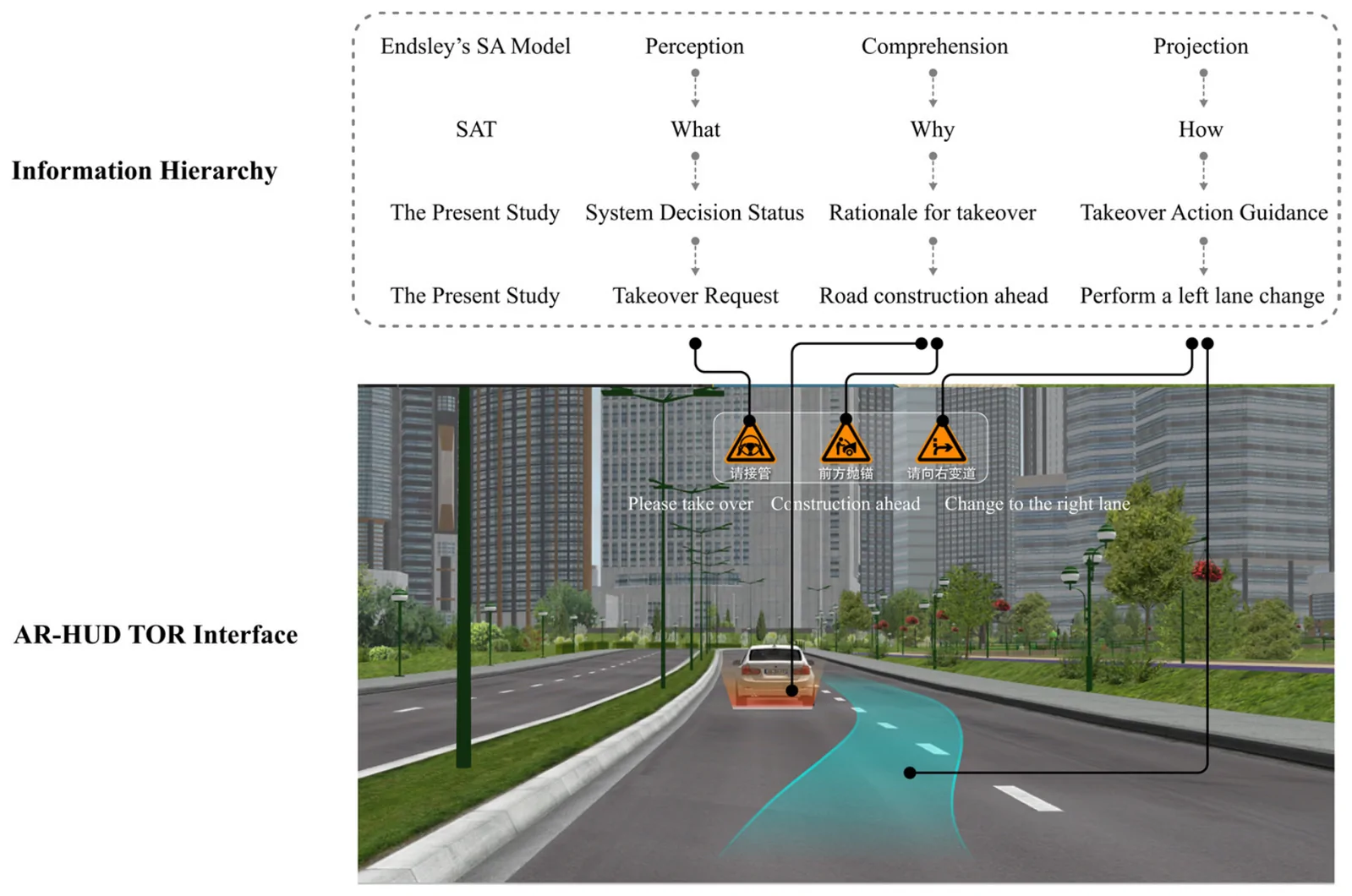
Design of the SAT-based AR-HUD takeover request (TOR) interface. The orange highlighting of the preceding target vehicle provides a visual warning, while the cyan trajectory line indicates the guided driving path.

All graphical elements were created using Adobe Illustrator 2020 (Adobe Inc., San Jose, CA, USA), and dynamic visual effects were produced using Adobe After Effects 2024 before being integrated into the driving simulation environment. To ensure experimental consistency, all interface conditions employed identical graphical content, icon design, color scheme, and layout. Only the presentation form (static vs. dynamic) and presentation timing (concurrent vs. progressive) were manipulated across the experimental conditions. The design of the TOR interface and the corresponding interface prototype are shown in Figure 2.

#### 2.3.2. Presentation Form

(1) Static presentation

**Figure 3:**
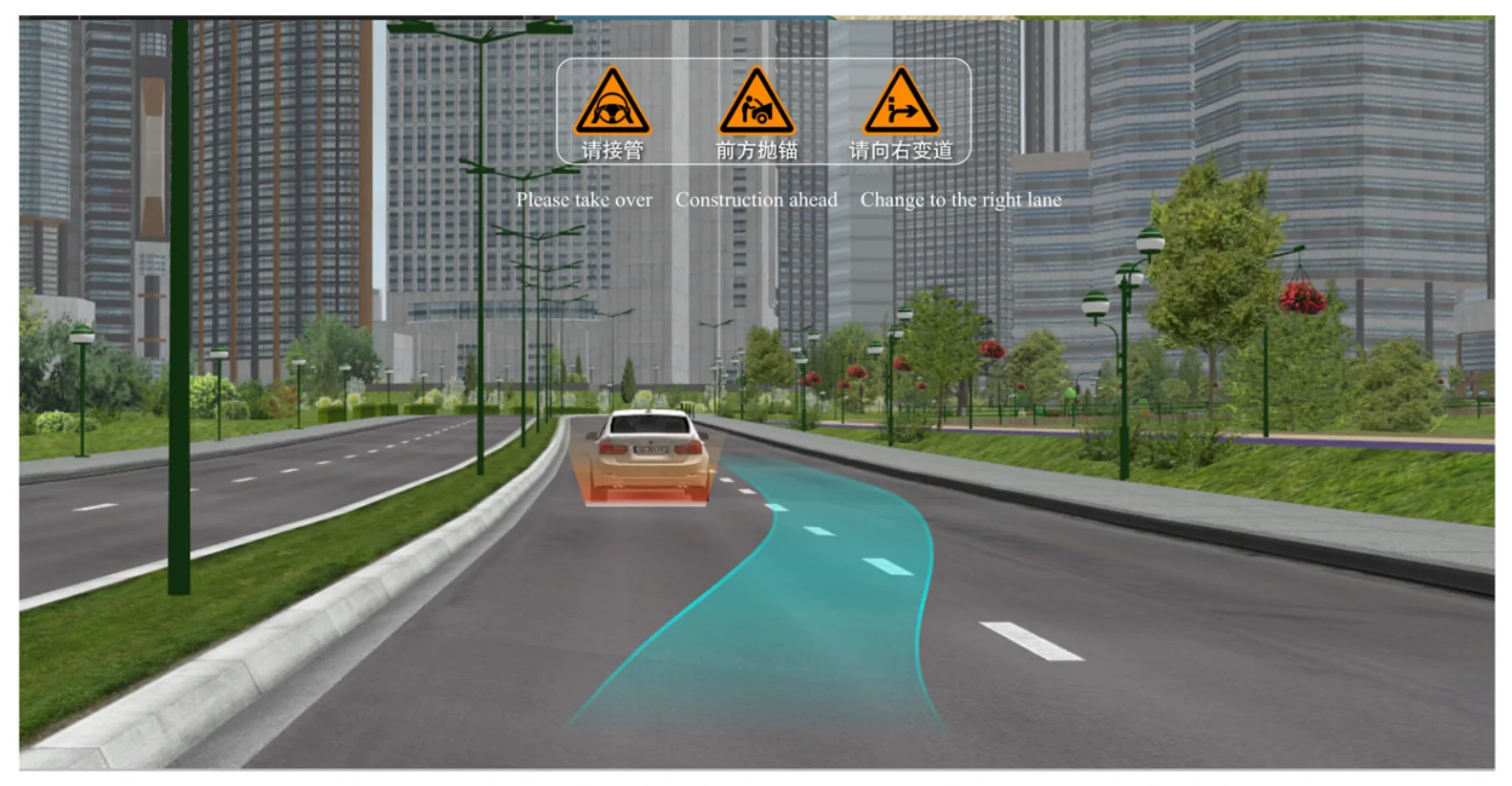
Static presentation interface in the baseline condition. The orange highlighting of the preceding target vehicle provides a visual warning, while the cyan trajectory line indicates the guided driving path.

As the baseline condition, static warning icons, textual information, and fixed trajectory lines were used to present system status and environmental information (Figure 3). Once presented, each visual element in the static condition remained visually unchanged for the remainder of the takeover-information interval.

(2) Dynamic presentation

Based on the static condition, dynamic visual features were introduced to enhance attentional guidance across three visual channels: icons, text, and trajectory lines.

**Figure 4:**
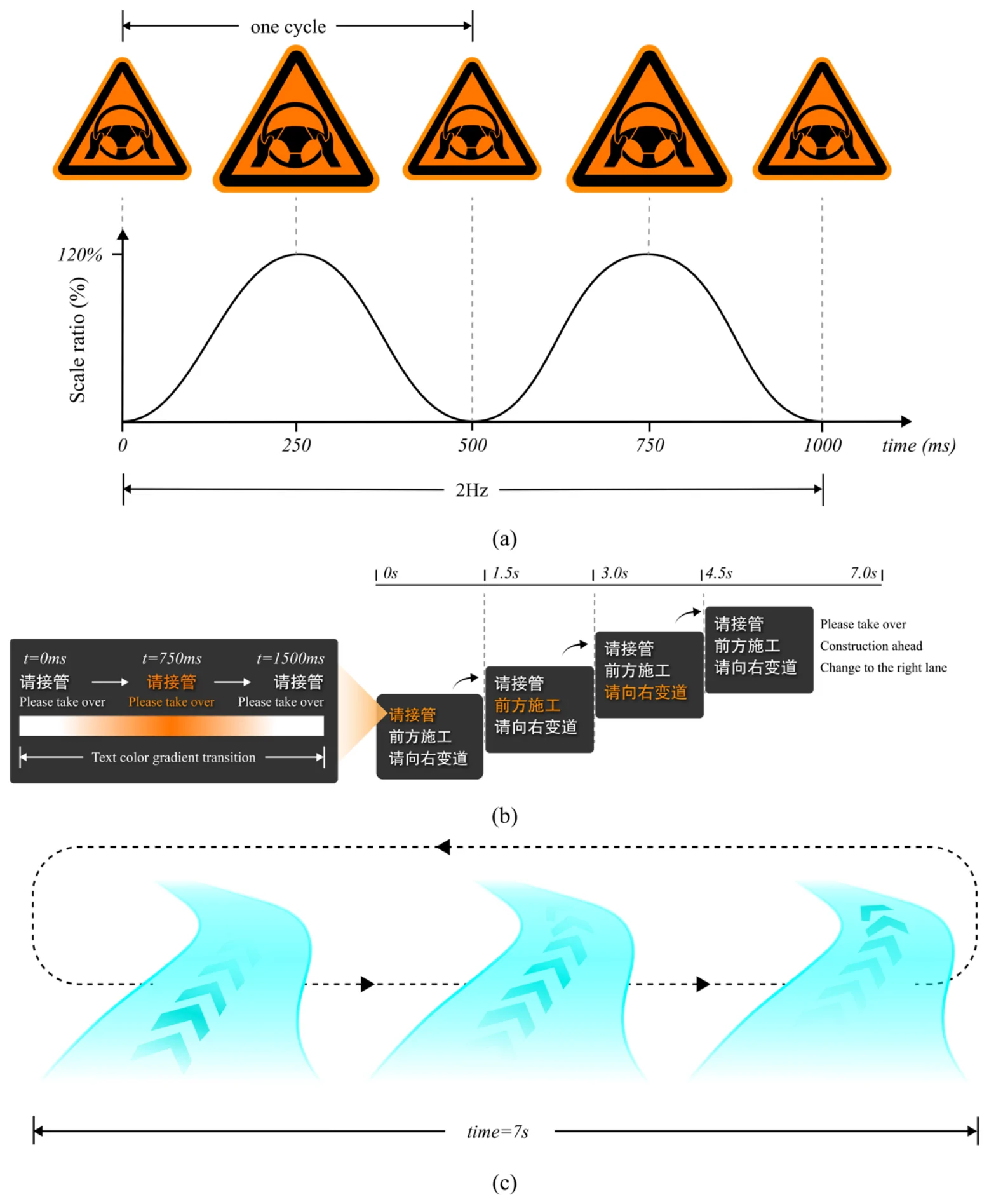
Dynamic visual presentation design of the takeover interface: (**a**) pulsing warning icon (100–120% scale, 2 Hz); (**b**) progressive text presentation using a color gradient across the What–Why–How information hierarchy; (**c**) dynamic trajectory animation, with cyan chevrons indicating the planned driving direction. Black dashed arrows indicate temporal progression between successive animation states.

- Dynamic warning icons employed a pulsing scale animation, oscillating between 100% and 120% of their original size at 2 Hz following the onset of the takeover request (TOR) (Figure 4a) [34].
- Dynamic text information adopted a color-gradient transition from a neutral color to a warning color (Figure 4b) to increase visual salience while maintaining text readability.
- Dynamic trajectory lines incorporated a continuous flow animation following the planned driving path (Figure 4c), providing dynamic guidance consistent with the vehicle’s intended trajectory.

#### 2.3.3. Presentation Timing

(1) Concurrent presentation

**Figure 5:**
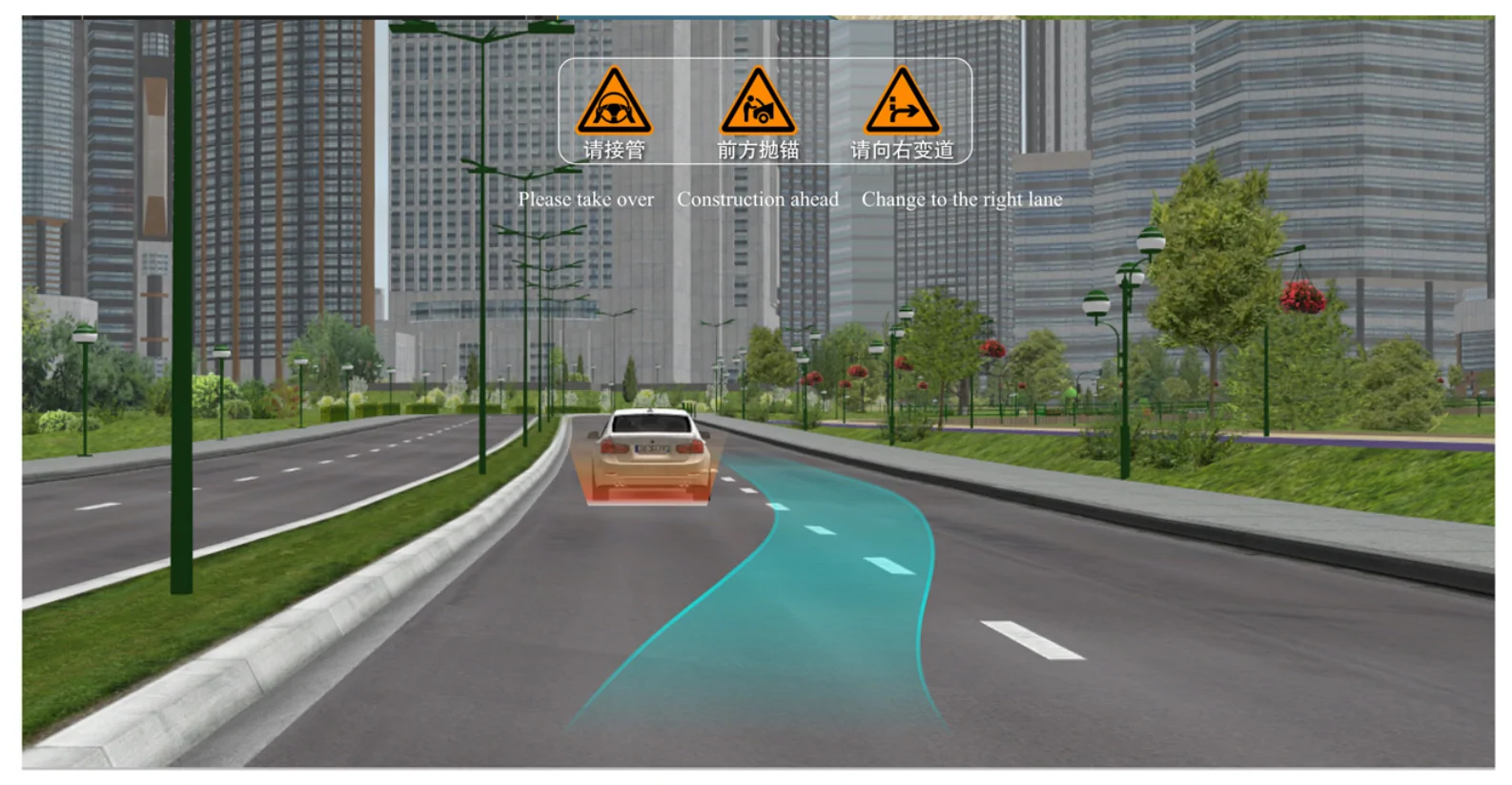
Concurrent presentation strategy of takeover information. The orange highlighting of the preceding target vehicle provides a visual warning, while the cyan trajectory line indicates the guided driving path.

As shown in Figure 5, at the moment of the takeover request (TOR), all layers of contextual information (i.e., environmental risk, takeover reason, and behavioral guidance) were simultaneously presented.

(2) Progressive presentation

**Figure 6:**
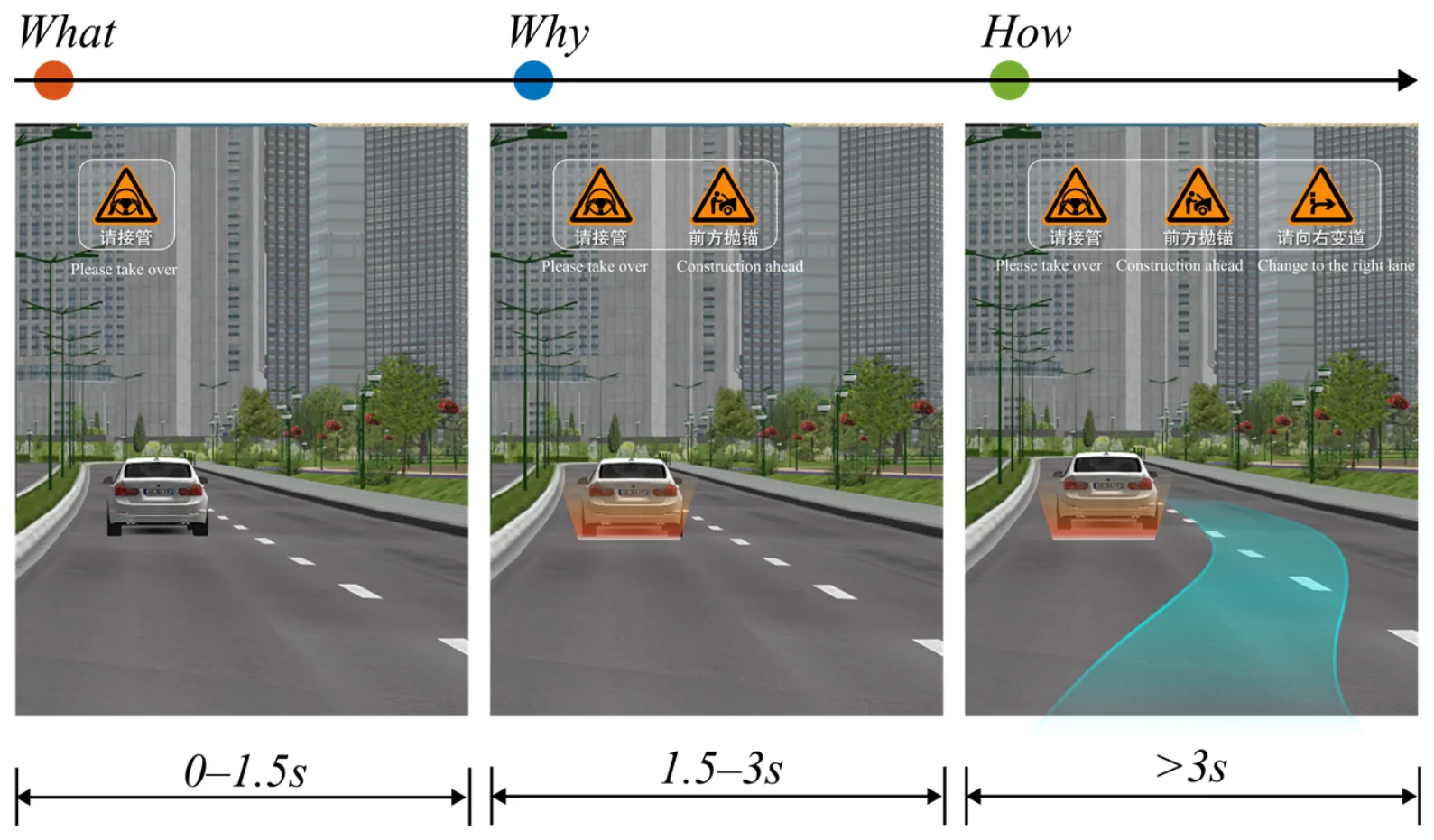
Progressive presentation strategy of takeover information. The orange highlighting of the preceding target vehicle provides a visual warning, while the cyan trajectory line indicates the guided driving path.

Based on drivers’ natural cognitive processing sequence, a progressive information disclosure strategy was developed in this study (see Figure 6) [35]. The strategy follows a strict temporal structure to guide different levels of cognitive processing.

- Stage 1 (0–1.5 s): The first stage presented only the basic takeover request (TOR), corresponding to the “What” information layer. The interface included warning icons and a textual prompt (e.g., “Please take over”), providing the initial takeover notification.
- Stage 2 (1.5–3.0 s): The second stage introduced information explaining the cause of the takeover request, corresponding to the “Why” information layer. Additional elements included hazard icons, explanatory text (e.g., “Vehicle breakdown ahead”), and annotations identifying the external hazard.
- Stage 3 (3.0–7.0 s): The final action guidance (“How” level) includes trajectory lines and text instructions (such as “Please change lanes to the right”). This stage is used to present recommended actions and their corresponding visual guidance.

#### 2.3.4. Experimental Conditions

**Figure 7:**
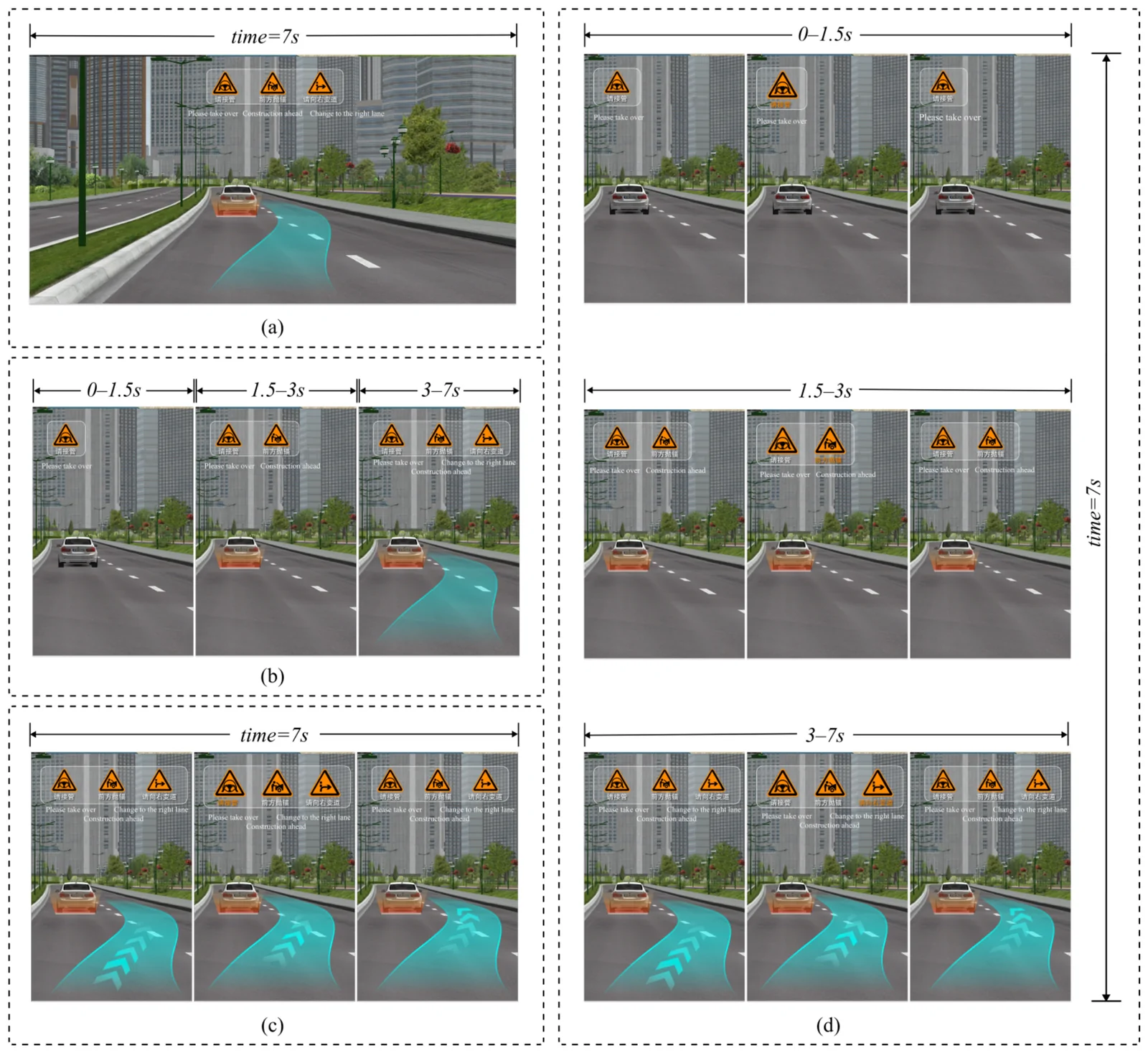
Full-factorial combinations of presentation form and presentation timing for the takeover interface in the vehicle breakdown scenario: (**a**) Static-concurrent presentation; (**b**) static-progressive presentation; (**c**) dynamic-concurrent presentation; (**d**) dynamic-progressive presentation. The orange highlighting of the preceding target vehicle provides a visual warning, while the cyan trajectory line indicates the guided driving path.

The combinations of the two levels of presentation form and presentation timing resulted in the final experimental stimuli. Using the vehicle breakdown scenario as an example, Figure 7 presents the high-fidelity takeover interfaces under the four experimental conditions, together with their temporal evolution throughout the takeover process.

### 2.4. Experimental Procedure

**Figure 8:**
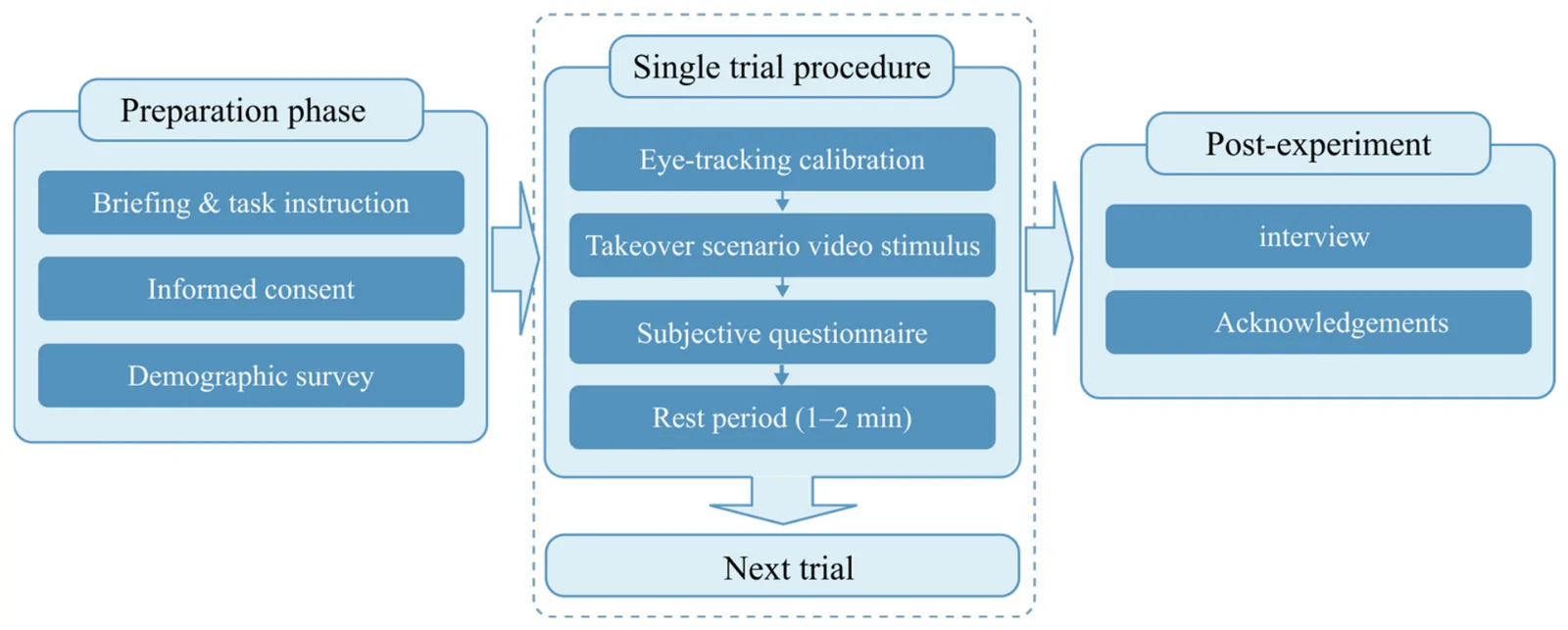
Overview of the experimental procedure.

The overall experimental procedure is illustrated in Figure 8. Upon arrival at the laboratory, participants received a standardized briefing on the experimental tasks and procedures. After confirming their understanding of the study, they provided written informed consent and completed a demographic questionnaire. Next, a calibration procedure was performed. Participants were instructed to maintain a comfortable seated posture and an appropriate viewing distance throughout the experiment. A standard nine-point calibration was then conducted to ensure the accuracy and reliability of the eye-tracking data. Calibration results are automatically evaluated by aSee Studio and expressed as calibration scores for the left and right eyes; calibration is considered successful when both scores are ≥90%. To minimize the impact of potential gaze drift during prolonged experiments, eye tracking calibration is performed again before the start of each trial to ensure the quality of eye tracking data collection for each trial. The formal experiment adopted a within-subject design, in which each participant completed eight automated driving takeover scenarios. To minimize potential order and carryover effects, condition order was controlled using a blocked 4 × 4 Latin-square counterbalancing design. Because the experiment comprised two automated driving takeover scenarios (vehicle breakdown and road construction), the four presentation conditions (static-concurrent, static-progressive, dynamic-concurrent, and dynamic-progressive) were counterbalanced independently within each scenario. Four sequences were generated per scenario, distributing each condition as evenly as possible across serial positions. The order of the two scenario blocks was also counterbalanced across participants.

In each trial, participants first viewed an automated driving video lasting approximately 14 s and were instructed to observe the driving scene naturally without performing any physical takeover actions. After each trial, participants completed a subjective evaluation questionnaire. A mandatory 1–2 min rest period was provided between consecutive trials to minimize visual fatigue. Eye-tracking data were continuously recorded throughout the experiment. The entire experimental session lasted approximately 15–20 min for each participant.

### 2.5. Measures

To evaluate the effects of presentation form and presentation timing on gaze behavior and subjective interface evaluations, objective eye-tracking measures were combined with subjective ratings.

#### 2.5.1. Eye-Tracking Measures and AOI Definition

Eye tracking provides a non-invasive technique for continuously recording gaze behavior during visual tasks. Four eye-movement measures were extracted to characterize fixation and visual-search patterns under the experimental conditions.

For the AOI-based analyses, the visual regions corresponding to What, Why, and How were operationalized as a single conceptual takeover-information area of interest (AOI), with the active spatial boundary defined according to the information visible during each temporal phase. The trajectory line, although retained as a visual path-guidance element in the stimulus, was not included in the AOI analysis because the AOI was designed to quantify drivers’ visual attention toward the takeover-information elements. AOI boundaries were defined in pixel coordinates relative to the 1920 × 1080 stimulus frame. AOI coordinates originated at the upper-left of the 1920 × 1080 stimulus frame and were proportionally scaled to the 3840 × 2160 display. In the concurrent condition, a fixed rectangular AOI encompassed all three information elements throughout the 7 s interval from the onset to the disappearance of the takeover information. In the progressive condition, the same conceptual takeover-information AOI was implemented using time-varying rectangular boundaries that encompassed the information elements visible during each phase. Specifically, the AOI covered What from 0 to 1.5 s, What and Why from 1.5 to 3.0 s, and What, Why, and How from 3.0 to 7.0 s. Keyframes were therefore defined at 0, 1.5, and 3.0 s relative to TOR onset. Regions corresponding to not-yet-visible elements were inactive until the respective elements appeared. Exact AOI keyframe timings, coordinates, annotated overlays, and corresponding stimulus videos are provided in the Supplementary Materials.

- Mean fixation duration (MFD) refers to the average duration of all fixations within the takeover-information AOI (unit: s). This metric is typically positively correlated with information complexity, visual-processing demands, or interface salience; a longer mean fixation duration generally indicates that participants devoted more sustained visual processing time to the target area [27,29].
- Mean saccade amplitude (MSA) refers to the average amplitude of all saccades recorded within the takeover-information AOI (unit: px). This measure reflects drivers’ visual search strategy. Larger saccade amplitudes are typically associated with global visual search, whereas smaller amplitudes indicate more localized visual exploration [36].
- Time to first fixation (TTFF) represents the elapsed time from TOR onset (t_0_ = 0 s) to the participant’s first fixation within the takeover-information AOI boundary active at that time (unit: s). The takeover-information AOI encompassed the What, Why, and How visual components, with a fixed boundary in the concurrent condition and phase-specific active boundaries in the progressive condition. A single TTFF value was calculated per trial without separate component-level calculations. TTFF reflects the efficiency of initial visual attention reallocation, with shorter values indicating that the takeover interface captured the driver’s attention more rapidly upon TOR onset [11].
- Fixation count (FC) refers to the total number of fixations within the takeover-information AOI. Higher fixation counts generally indicate more frequent information acquisition and greater visual-search or information-processing demands [27,29].

#### 2.5.2. Eye-Tracking Data Quality Assessment

To evaluate the reliability of eye-tracking data, the tracking ratio provided by the aSee Studio system was calculated for each participant and trial. The tracking ratio was defined as the proportion of valid gaze samples relative to the total recorded gaze samples during each trial. Samples missing because of blinks or temporary loss of pupil or corneal-reflection signals were classified as technical tracking loss and treated as missing samples without interpolation. Technical tracking loss was distinguished from AOI non-fixation, which indicated valid gaze recording but no fixation satisfying the I-VT criteria within the takeover-information AOI boundary active at that time during the 7 s interval.

Three participants were excluded during preprocessing because their tracking ratios were below 75%. Following these exclusions, the final dataset consisted of 21 participants for subsequent eye-tracking analyses. The overall mean tracking ratio across all retained participants and trials was 86.26%, corresponding to a data-loss rate of 13.74%. At the participant level, mean tracking ratios ranged from 75.78% to 95.88%, with corresponding data-loss rates ranging from 4.12% to 24.22%. At the trial level, mean tracking ratios ranged from 80.96% to 91.46% across the eight experimental trials, with corresponding data-loss rates ranging from 8.54% to 19.04%. When aggregated by experimental condition, the mean tracking ratios were 86.17%, 89.54%, 81.11%, and 88.34% for the static-concurrent, static-progressive, dynamic-concurrent, and dynamic-progressive conditions, respectively. The corresponding data-loss rates were 13.83%, 10.46%, 18.89%, and 11.66%. Collectively, these results indicate that the retained eye-tracking data were of acceptable quality across participants, trials, and experimental conditions. All 168 retained trials contained at least one valid fixation within the takeover-information AOI that was active at the corresponding time. Therefore, no trial required exclusion or imputation because of AOI non-fixation.

#### 2.5.3. Subjective Evaluation Instruments

After each scenario trial, participants evaluated the interface presentation strategy using subjective rating scales. Perceived usability and intention comprehension were assessed using five- and seven-point Likert scales, respectively. Interface acceptance was assessed using a five-point semantic differential scale. The individual measures are summarized in Table 1.

- Perceived usability was assessed using a study-specific exploratory five-item measure informed by the System Usability Scale (SUS) framework [37]. Considering the characteristics of AR-HUD takeover displays, five items were selected and adapted from the original SUS framework to evaluate clarity, readability, comprehensibility, information integration, and complexity. The SUS item-scoring logic was retained, with responses converted to a 0–100 scale to facilitate interpretation. Each item was rated on a five-point Likert scale. Positive items were recoded from 0 to 4 by subtracting 1 from the raw response, and the negatively worded complexity item was reverse-coded from 0 to 4 by subtracting the raw response from 5. The five recoded item scores were summed and multiplied by 5 to produce a usability score ranging from 0 to 100, with higher scores indicating greater perceived usability. This linear rescaling was used solely to facilitate interpretation, with 0 and 100 representing the minimum and maximum possible scores, respectively.
- Acceptance was measured using the Van der Laan Acceptance Scale (VDLAS). Nine bipolar adjective pairs assessed the dimensions of usefulness and satisfaction. Each item was coded from −2 to 2, with 2 representing the positive endpoint, 0 the midpoint, and −2 the negative endpoint. The overall acceptance score was calculated as the mean of the nine items, with higher scores indicating greater acceptance.
- Intention comprehension assessed how effectively the interface supported participants’ understanding of the system’s intentions. The three items measured the clarity of vehicle intentions, logical consistency, and predictability of vehicle behavior. The mean item score was calculated, ranging from 1 to 7, with higher scores indicating better intention comprehension.

## 3. Results

To evaluate the effectiveness of the counterbalancing procedure, potential order effects were examined. Participants were grouped according to scenario-block order: vehicle breakdown first or road construction first. Mann–Whitney U tests revealed no significant differences between the groups in mean fixation duration (MFD), mean saccade amplitude (MSA), time to first fixation (TTFF), or fixation count (FC; all *p* > 0.05). Kruskal–Wallis tests conducted separately for each scenario likewise showed no significant effects of the Latin-square sequence on any eye-tracking measure (all *p* > 0.05). Thus, no statistical evidence indicated that scenario order or condition order affected the eye-tracking results.

### 3.1. Eye-Tracking Measures

Four eye-tracking measures were analyzed to assess the effects of presentation form (static vs. dynamic) and presentation timing (concurrent vs. progressive) on gaze behavior during automated driving takeover scenarios. Shapiro–Wilk tests indicated no significant deviations from normality for any measure under any experimental condition. Accordingly, separate 2 × 2 repeated-measures analyses of variance (ANOVAs) were conducted to examine the main effects of presentation form and presentation timing and their interaction. Significant main effects were followed by pairwise comparisons based on estimated marginal means, whereas significant interactions were examined using simple-effects analyses. Bonferroni corrections were applied to multiple comparisons. Statistical significance was set at α = 0.05, and effect sizes were reported as partial eta squared ($\eta_{p}^{2}$).

#### 3.1.1. Mean Fixation Duration (MFD)

**Figure 9:**
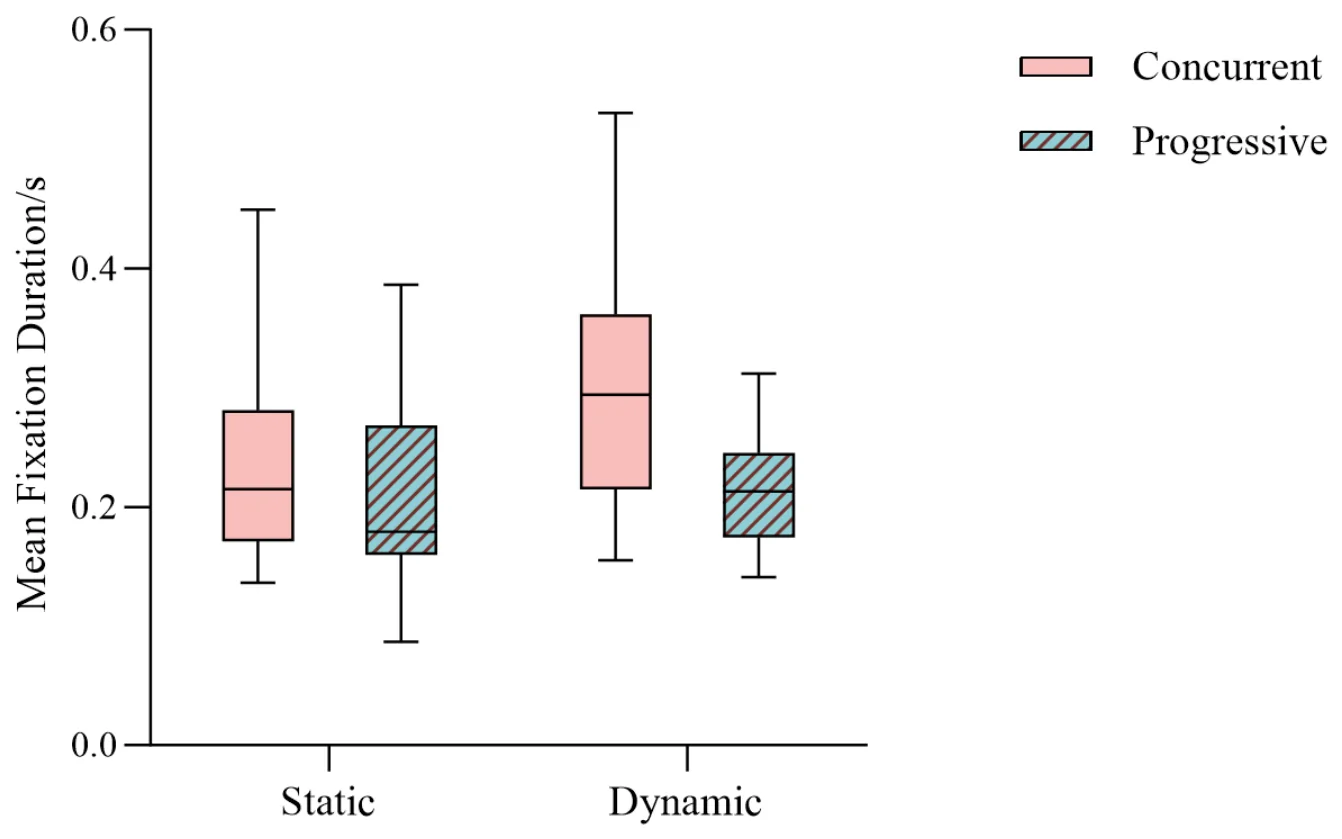
Boxplots of mean fixation duration (MFD) under the four experimental conditions.

As shown in Figure 9, the ANOVA revealed a significant main effect of presentation form on MFD (F(1, 20) = 7.051, *p* = 0.015, $\eta_{p}^{2}$ = 0.261). MFD was significantly longer under dynamic presentation (M ± SD = 0.254 ± 0.088) than under static presentation (M ± SD = 0.219 ± 0.080). Presentation timing also had a significant main effect (F(1, 20) = 12.589, *p* = 0.002, $\eta_{p}^{2}$ = 0.386). MFD was significantly longer under concurrent presentation (M ± SD = 0.265 ± 0.093) than under progressive presentation (M ± SD = 0.208 ± 0.066). The interaction between presentation form and presentation timing was not significant (F(1, 20) = 2.654, *p* = 0.119, $\eta_{p}^{2}$ = 0.117).

#### 3.1.2. Mean Saccade Amplitude (MSA)

Presentation form had no significant main effect on MSA (F(1, 20) = 0.196, *p* = 0.663, $\eta_{p}^{2}$ = 0.010). By contrast, presentation timing had a significant main effect (F(1, 20) = 11.140, *p* = 0.003, $\eta_{p}^{2}$ = 0.358). MSA was significantly smaller under progressive presentation (M ± SD = 142.844 ± 44.224) than under concurrent presentation (M ± SD = 181.988 ± 48.597).

**Figure 10:**
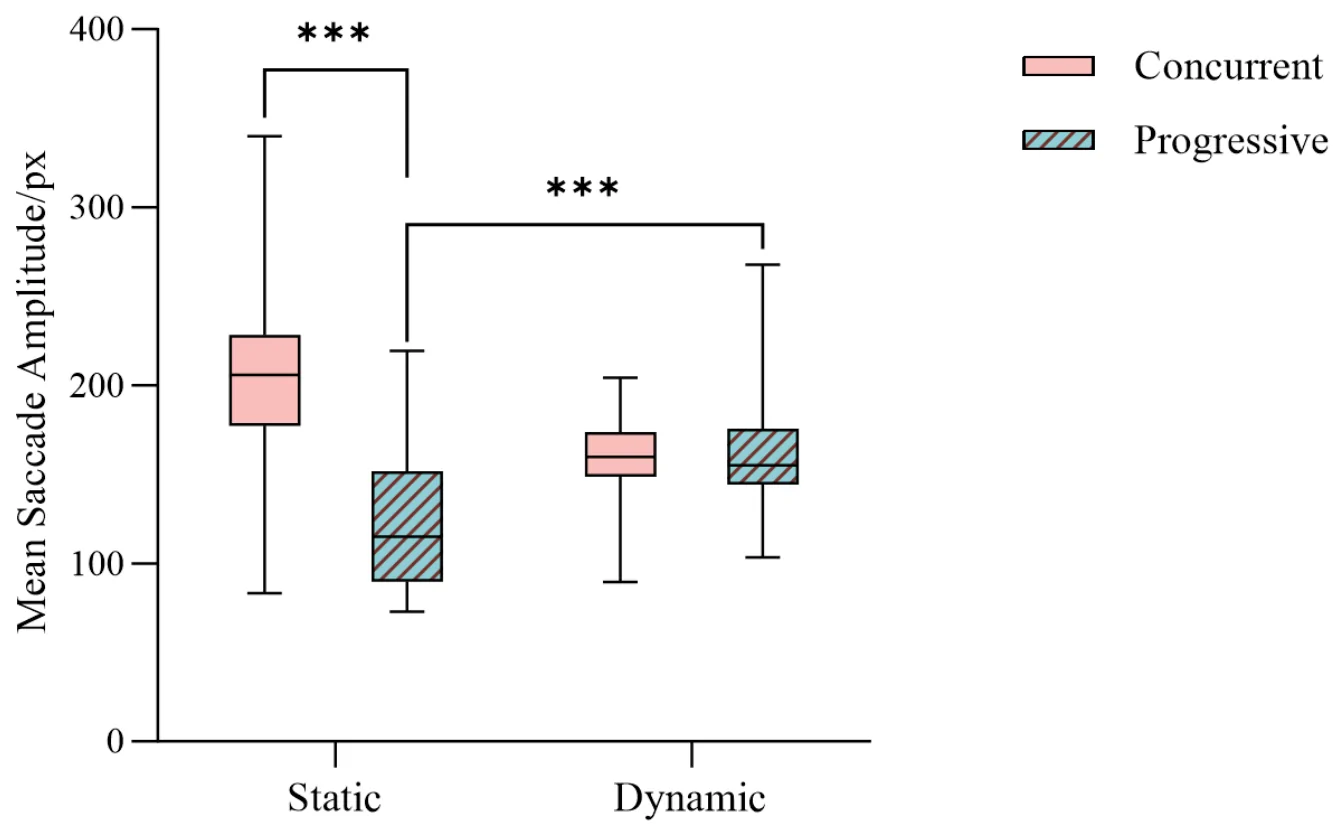
Boxplots of mean saccade amplitude (MSA) under the four experimental conditions. (*** *p* ≤ 0.001).

The interaction between presentation form and presentation timing was significant (F(1, 20) = 76.210, *p* < 0.001, $\eta_{p}^{2}$ = 0.792). Bonferroni-adjusted simple-effects analyses are presented in Figure 10. Under static presentation, MSA was significantly smaller for progressive presentation (M ± SD = 122.360 ± 40.041) than for concurrent presentation (M ± SD = 205.690 ± 54.068, F(1, 20) = 32.007, *p* < 0.001, $\eta_{p}^{2}$ = 0.615). Under dynamic presentation, MSA did not differ significantly between the concurrent (M ± SD = 158.286 ± 27.175) and progressive conditions (M ± SD = 163.327 ± 39.051, F(1, 20) = 0.232, *p* = 0.635, $\eta_{p}^{2}$ = 0.011). Under progressive presentation, MSA was significantly smaller for static presentation than for dynamic presentation (F(1, 20) = 35.432, *p* < 0.001, $\eta_{p}^{2}$ = 0.639).

#### 3.1.3. Time to First Fixation (TTFF)

Presentation form had no significant main effect on TTFF (F(1, 20) = 0.172, *p* = 0.683, $\eta_{p}^{2}$ = 0.009). Presentation timing had a significant main effect (F(1, 20) = 34.533, *p* < 0.001, $\eta_{p}^{2}$ = 0.633). TTFF was significantly shorter under progressive presentation (M ± SD = 0.602 ± 0.526) than under concurrent presentation (M ± SD = 1.205 ± 0.627).

**Figure 11:**
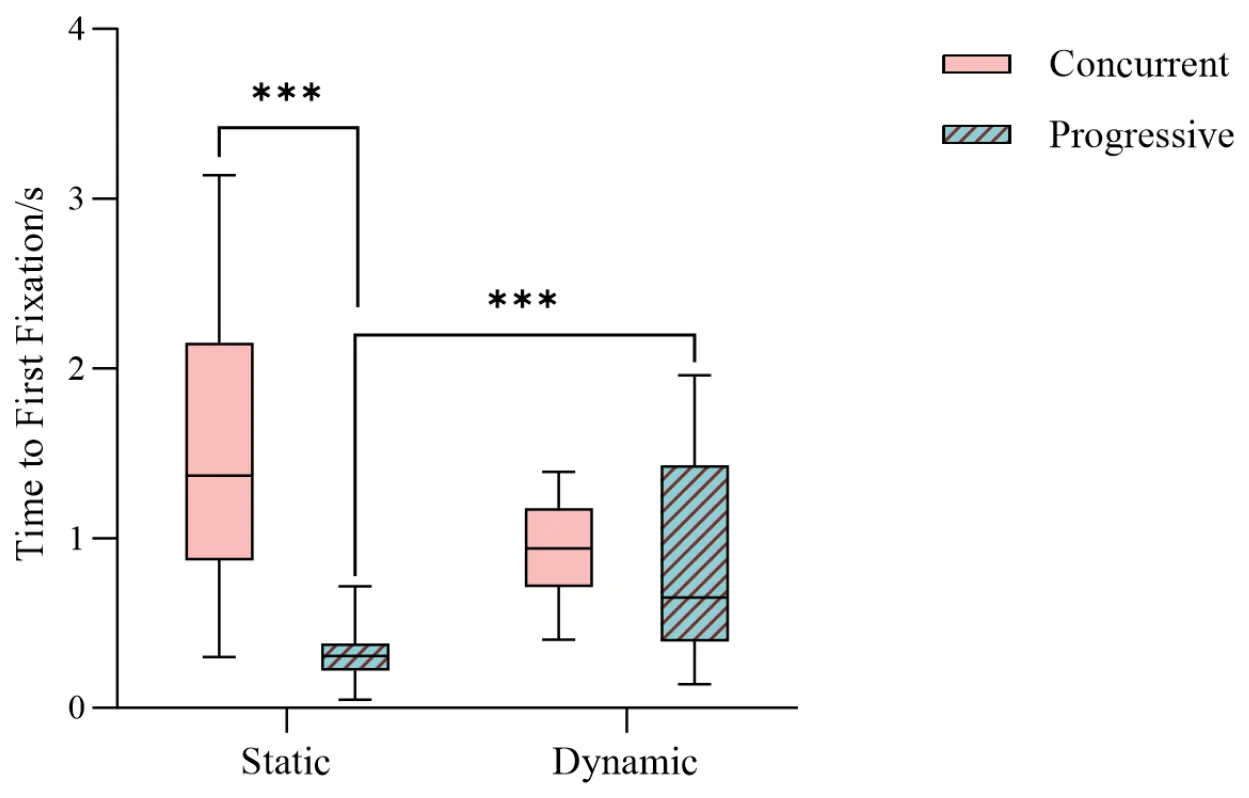
Boxplots of time to first fixation (TTFF) under the four experimental conditions. (*** *p* ≤ 0.001).

The interaction between presentation form and presentation timing was significant (F(1, 20) = 32.208, *p* < 0.001, $\eta_{p}^{2}$ = 0.617). Bonferroni-adjusted simple-effects analyses are shown in Figure 11. Under static presentation, TTFF was significantly shorter for progressive presentation (M ± SD = 0.283 ± 0.036) than for concurrent presentation (M ± SD = 1.477 ± 0.755, F(1, 20) = 53.729, *p* < 0.001, $\eta_{p}^{2}$ = 0.729). Under dynamic presentation, TTFF did not differ significantly between the concurrent (M ± SD = 0.933 ± 0.282) and progressive conditions (M ± SD = 0.921 ± 0.593, F(1, 20) = 0.009, *p* = 0.926, $\eta_{p}^{2}$ < 0.001). Under progressive presentation, TTFF was significantly shorter for static presentation than for dynamic presentation (F(1, 20) = 24.515, *p* < 0.001, $\eta_{p}^{2}$ = 0.551).

#### 3.1.4. Fixation Count (FC)

**Figure 12:**
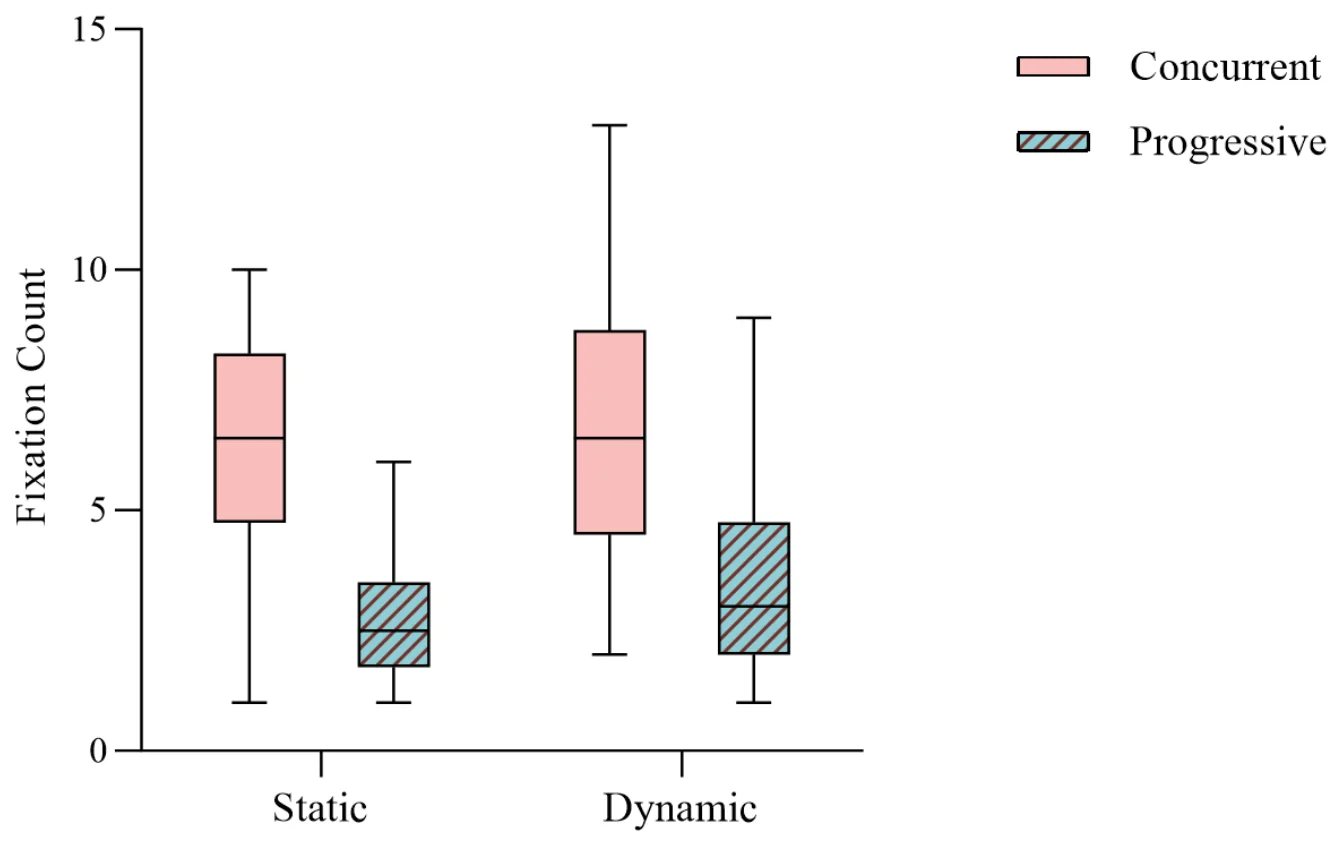
Boxplots of fixation count (FC) under the four experimental conditions.

As shown in Figure 12, neither the main effect of presentation form (F(1, 20) = 1.789, *p* = 0.196, $\eta_{p}^{2}$ = 0.082), nor its interaction with presentation timing was significant (F(1, 20) = 0.128, *p* = 0.724, $\eta_{p}^{2}$ = 0.006). Presentation timing had a significant main effect (F(1, 20) = 60.616, *p* < 0.001, $\eta_{p}^{2}$ = 0.752). Progressive presentation produced significantly fewer fixations (M ± SD = 3.155 ± 1.716) than concurrent presentation (M ± SD = 6.536 ± 2.572).

**Figure 13:**
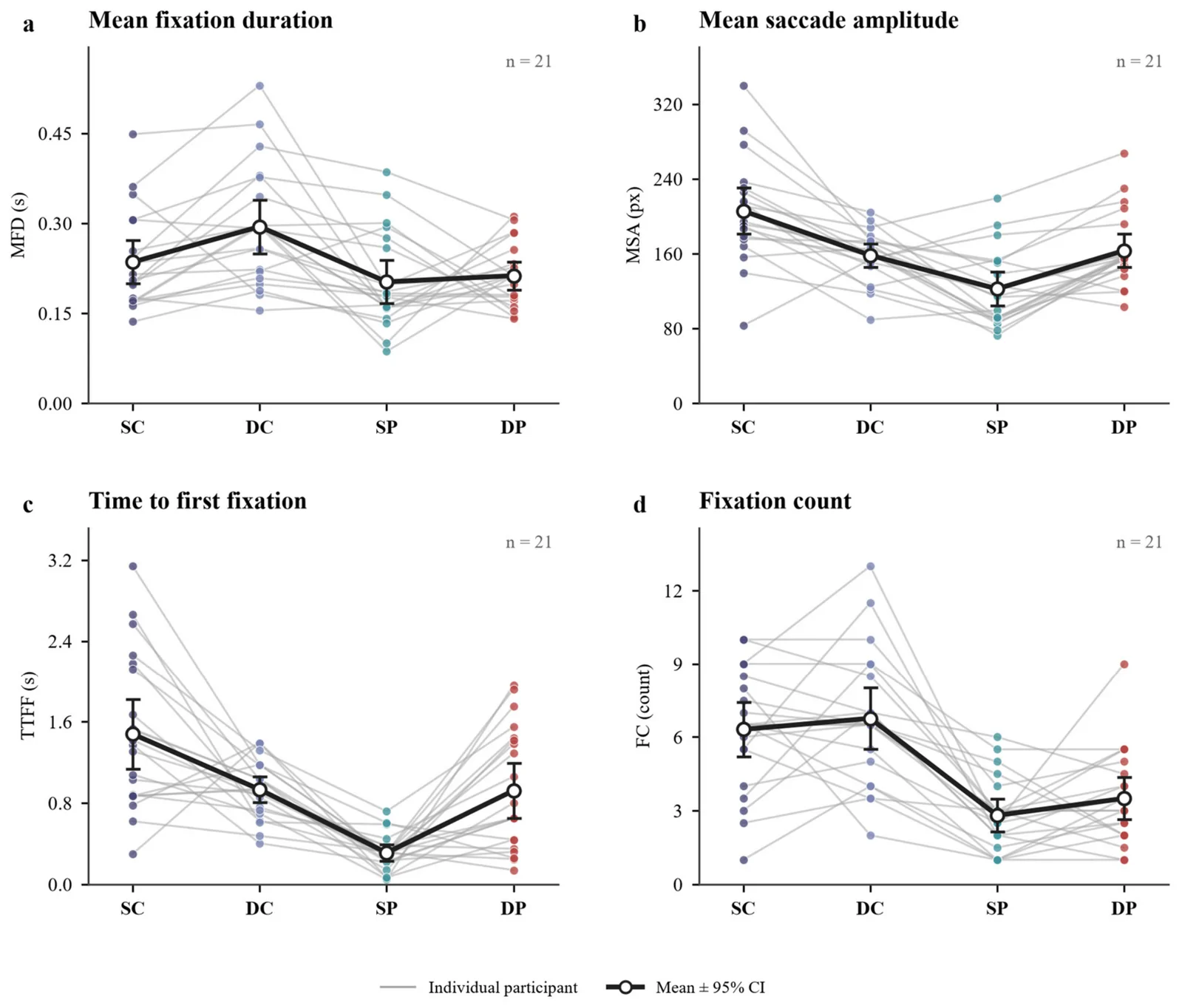
Individual variability in eye-tracking measures across four AR-HUD information presentation conditions. Gray lines connect observations from the same participant across conditions, and colored circles represent individual data. Thick black lines and open circles indicate group means, with error bars representing 95% confidence intervals (*n* = 21). SC, static-concurrent; DC, dynamic-concurrent; SP, static-progressive; DP, dynamic-progressive.

To further examine the consistency of the experimental effects across participants, spaghetti plots were used to visualize individual-level response patterns across the four experimental conditions (Figure 13). The gray lines represent individual participants’ trajectories across conditions, whereas the black lines indicate the group means with 95% confidence intervals. Overall, the individual trajectories were generally consistent with the statistical findings. Compared with concurrent presentation, progressive presentation showed lower values on the eye-tracking measures for most participants, with the most evident differences observed in the static-progressive condition. Although individual variability was present, the general direction of the observed differences was relatively consistent across participants.

#### 3.1.5. Heatmaps and Gaze Scanpaths

To illustrate gaze patterns across the four conditions, aggregated heatmaps and superimposed participant-level scanpaths were generated in aSee Studio using data from all valid participants. The heatmaps summarized fixation density across participants. For the scanpaths, each participant’s sequence was generated separately and overlaid on the same reference frame over the 7 s interval. Colors identified participants, while numbers and connecting lines indicated fixation order and consecutive transitions within each participant. No temporal averaging or cross-participant connections were performed. These visualizations were used descriptively to complement the quantitative results.

**Figure 14:**
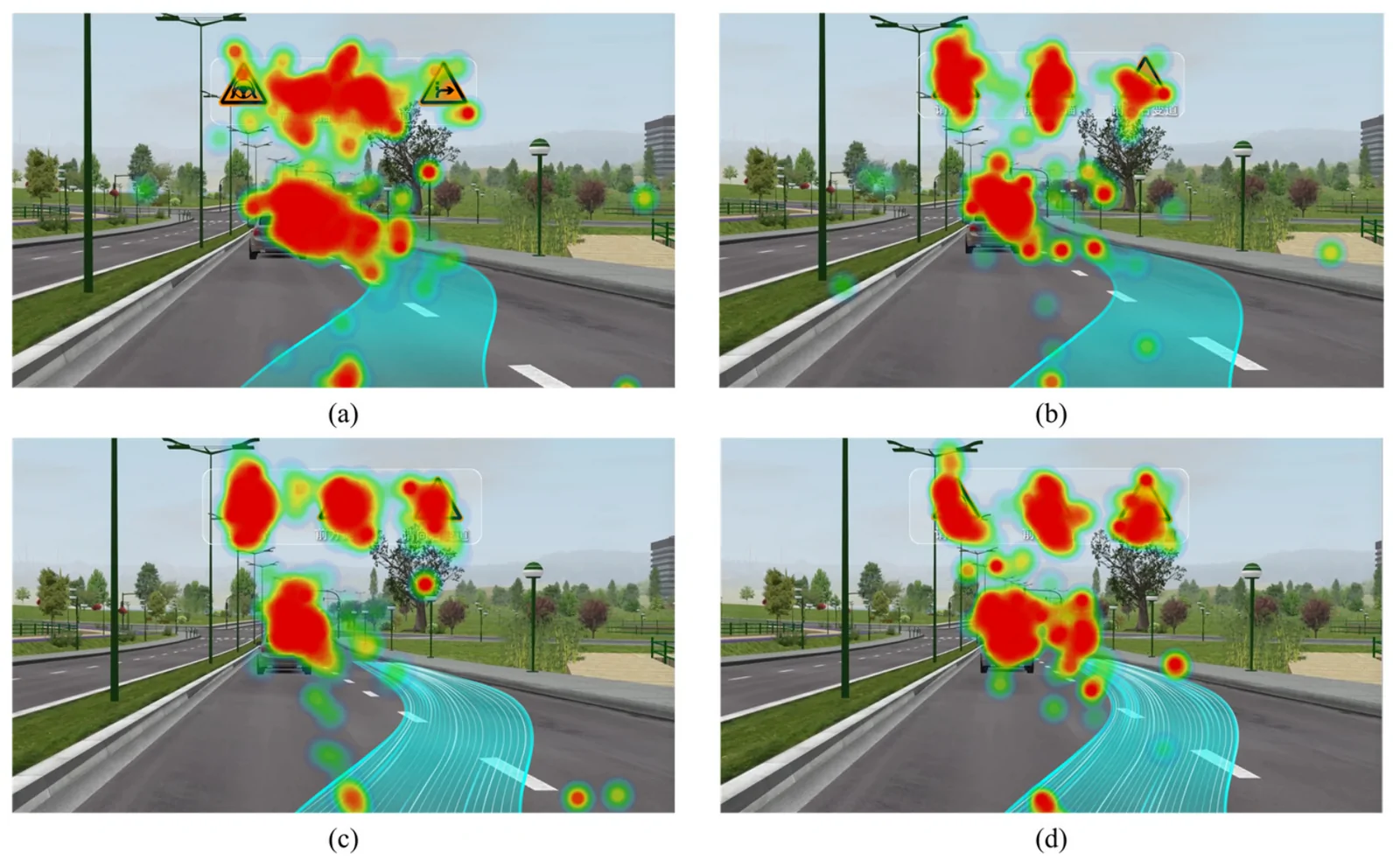
Gaze heatmaps of visual attention distribution under the four experimental conditions: (**a**) static-concurrent; (**b**) static-progressive; (**c**) dynamic-concurrent; (**d**) dynamic-progressive. Colored regions indicate areas viewed by participants; red indicates longer fixation duration, whereas green indicates shorter fixation.

As shown in Figure 14, the two presentation timings produced distinct gaze distributions. Under the concurrent presentation condition (Figure 14a,c), fixation hotspots were widely distributed across the interface, indicating more dispersed visual attention. In contrast, under the progressive presentation condition (Figure 14b,d), fixation hotspots were highly concentrated within the target icon region, with a few fixations distributed outside the target region. The dynamic-progressive condition (Figure 14d) showed a larger and more intense high-density fixation region than the static-progressive condition (Figure 14b), consistent with the longer MFD observed under dynamic presentation.

**Figure 15:**
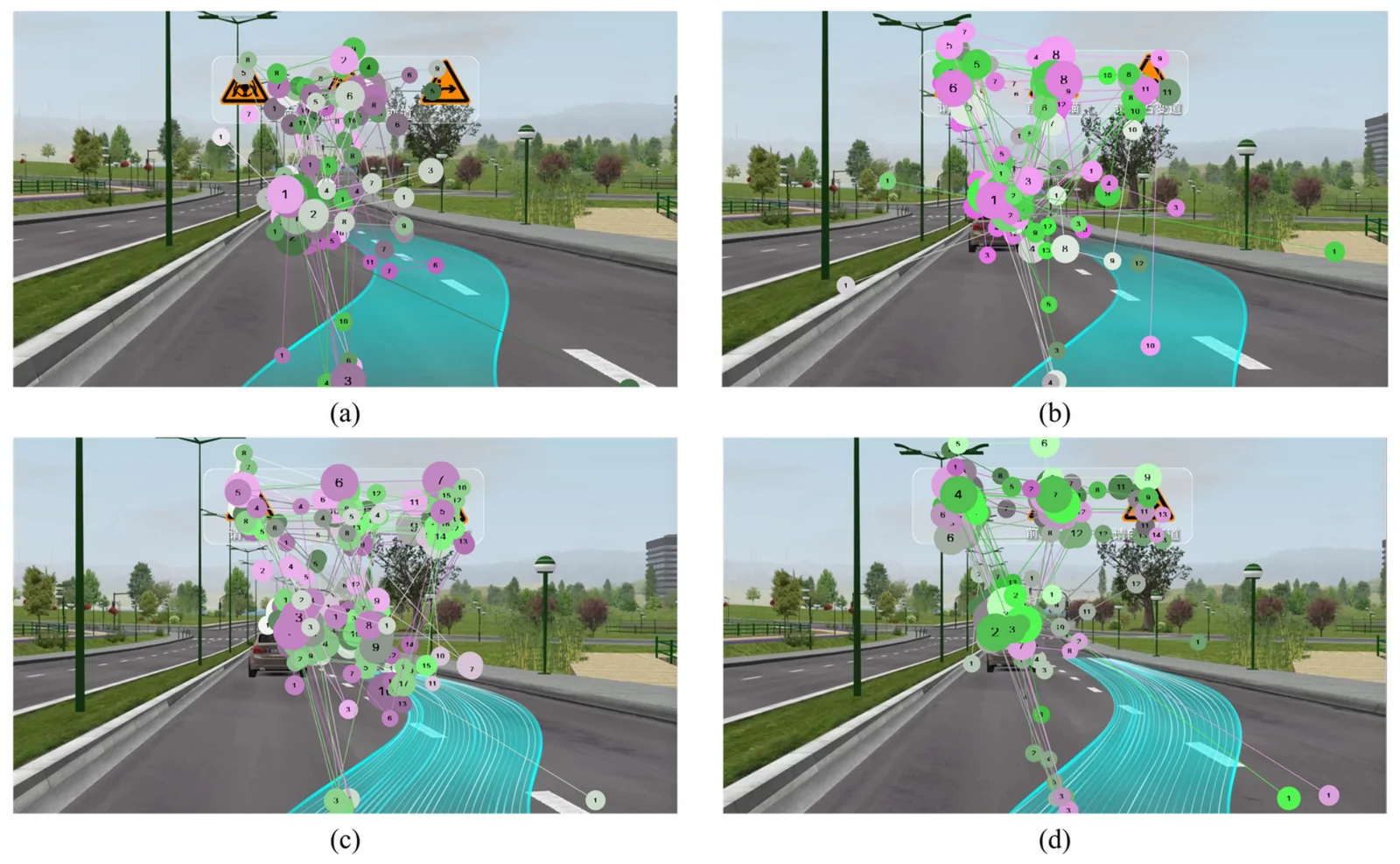
Superimposed participant-level gaze scanpaths under the four experimental conditions: (**a**) static-concurrent; (**b**) static-progressive; (**c**) dynamic-concurrent; (**d**) dynamic-progressive. Numbers within the fixation circles indicate the fixation sequence, and different colors distinguish individual participants.

As shown in Figure 15, gaze scanpaths under the concurrent presentation condition (Figure 15a,c) exhibited frequent transitions among the screen center, surrounding traffic environment, and target icons, indicating extensive visual scanning. In contrast, visual inspection of the superimposed participant-level scanpaths under progressive presentation (Figure 15b,d) suggested more direct gaze paths and fewer long-range within-participant transitions. This more orderly search pattern was descriptively consistent with the shorter TTFF observed under progressive presentation. Under the dynamic progressive condition (Figure 15d), the linear search pattern was preserved, while more fixation points and denser local trajectory clusters were observed within the target region than under the static-progressive condition (Figure 15b). This pattern indicated more sustained visual processing of the target information.

Overall, the qualitative gaze patterns corresponded to the quantitative eye-tracking results. Concurrent presentation showed more spatially dispersed fixations and more frequent gaze transitions across information elements, whereas progressive presentation showed more concentrated fixations and fewer transitions. Dynamic presentation was associated with denser target-region fixations and more repeated local transitions, consistent with its longer MFD. The heatmaps and scanpaths therefore provided descriptive visualizations of gaze behavior under the four presentation conditions.

### 3.2. Subjective Evaluation Measures

To comprehensively evaluate participants’ subjective evaluations of the different information presentation strategies, ratings of perceived usability, interface acceptance, and intention comprehension were collected. Shapiro–Wilk tests indicated that the subjective ratings satisfied the normality assumption. Separate two-way repeated-measures ANOVAs were therefore conducted for the three measures.

#### 3.2.1. Perceived Usability

Perceived usability was assessed using the study-specific exploratory five-item measure described in Section 2.5.3. The measure showed good internal consistency in the present sample (Cronbach’s α = 0.840), with an adequate Kaiser–Meyer–Olkin value (KMO = 0.832).

**Figure 16:**
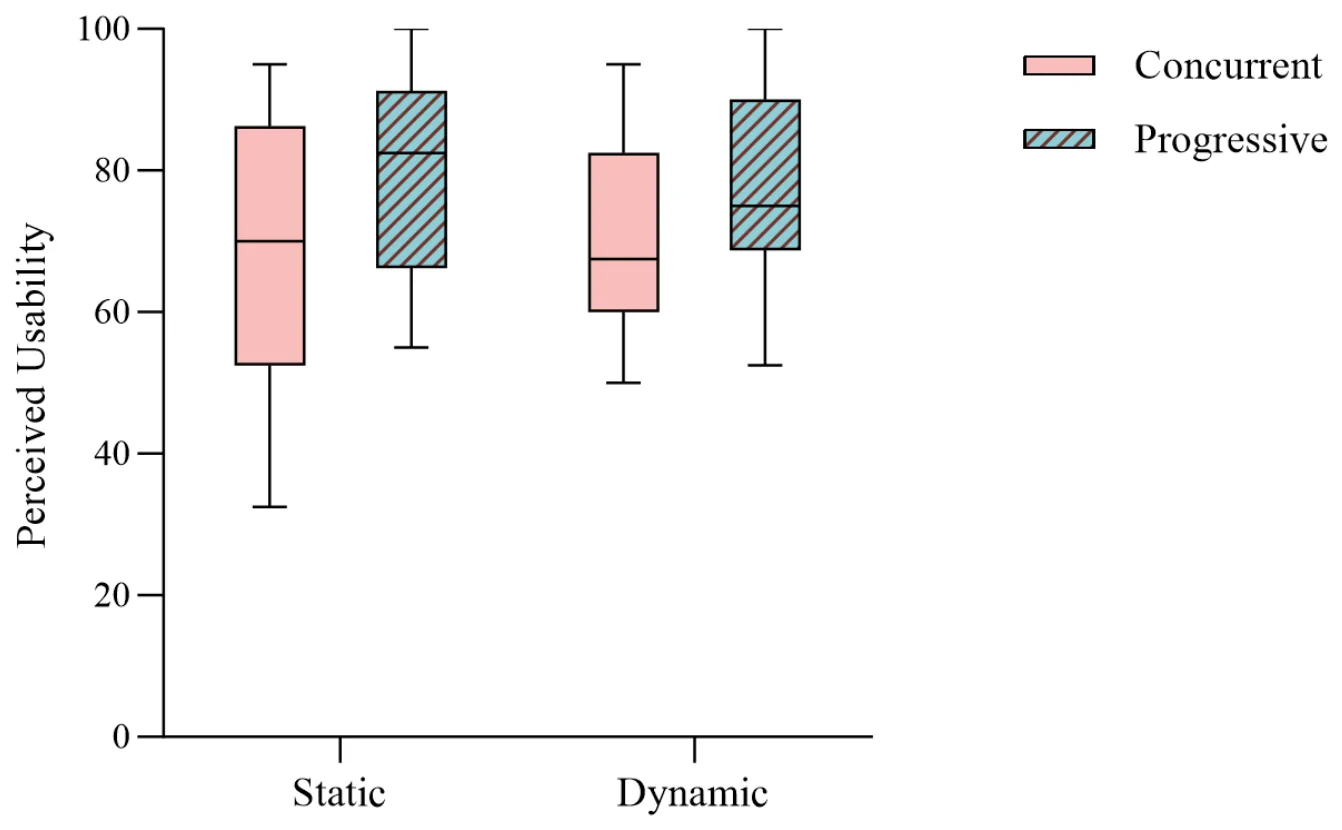
Boxplot of perceived usability scores under the concurrent and progressive display conditions.

As shown in Figure 16, the main effect of presentation form was not significant (F(1, 20) = 0.281, *p* = 0.602, $\eta_{p}^{2}$ = 0.014). The interaction between presentation form and presentation timing was also not significant (F(1, 20) = 1.478, *p* = 0.238, $\eta_{p}^{2}$ = 0.069). Presentation timing had a significant main effect (F(1, 20) = 8.805, *p* = 0.008, $\eta_{p}^{2}$ = 0.306). Perceived usability was significantly higher under progressive presentation (M ± SD = 78.274 ± 13.721) than under concurrent presentation (M ± SD = 69.881 ± 16.323).

#### 3.2.2. Interface Acceptance

The interface acceptance scale showed excellent internal consistency (Cronbach’s α = 0.961) and an adequate KMO value (KMO = 0.804). Neither presentation form (F(1, 20) = 0.051, *p* = 0.823, $\eta_{p}^{2}$ = 0.003) nor presentation timing had a significant main effect (F(1, 20) = 1.726, *p* = 0.204, $\eta_{p}^{2}$ = 0.079). Their interaction was also not significant (F(1, 20) = 0.039, *p* = 0.845, $\eta_{p}^{2}$ = 0.002).

**Figure 17:**
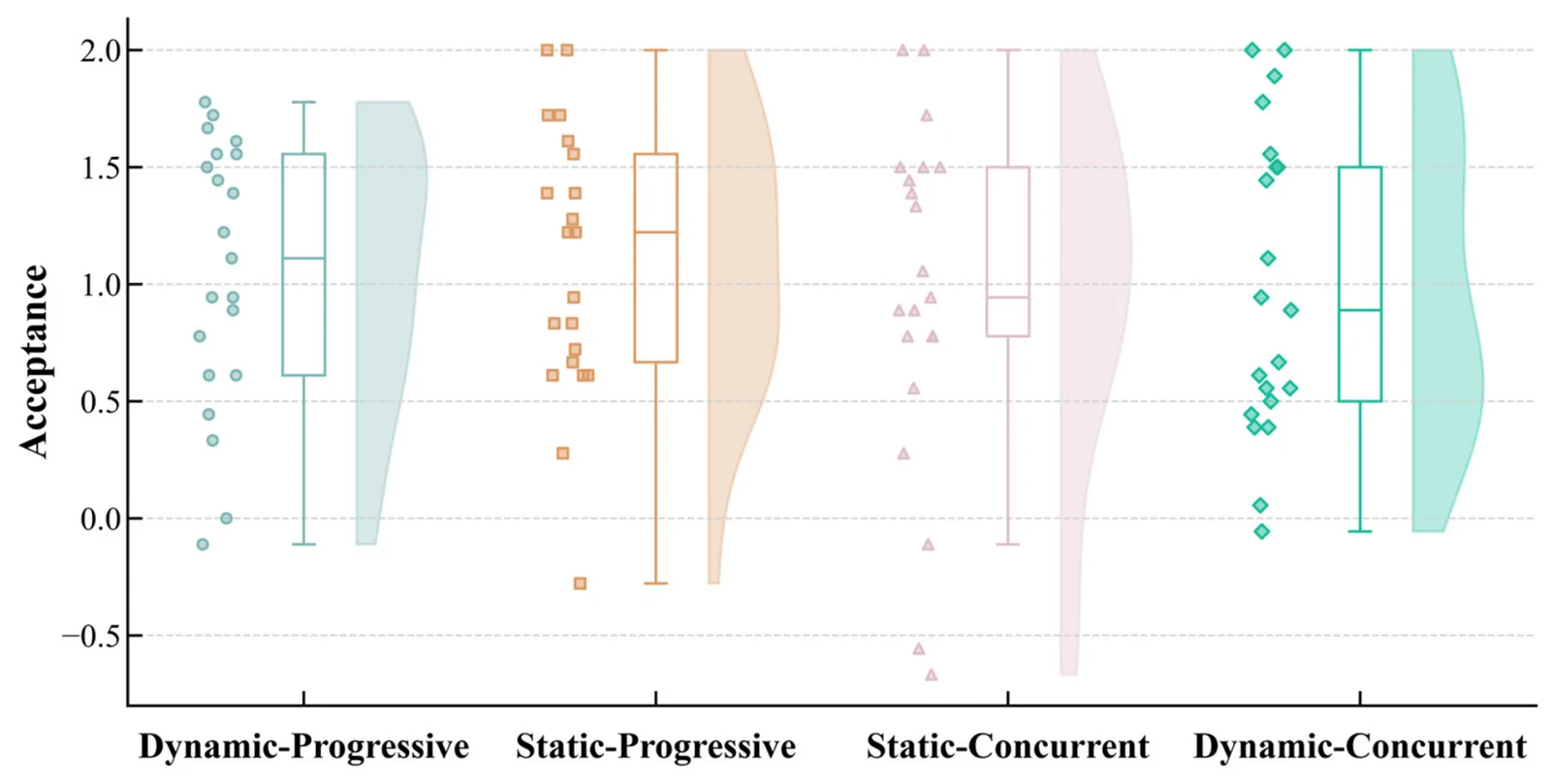
Interface acceptance scores under the four experimental conditions. Each point represents an individual observation; circles, squares, triangles, and diamonds represent the dynamic-progressive, static-progressive, static-concurrent, and dynamic-concurrent conditions, respectively. Boxplots indicate the median and interquartile range (IQR), while the half-violin plots illustrate the distribution of the data.

Descriptively, acceptance scores were slightly higher in both progressive conditions than in their corresponding concurrent conditions (Figure 17). The static-progressive condition produced the highest score (M ± SD = 1.077 ± 0.587), followed by dynamic-progressive (M ± SD = 1.063 ± 0.572), dynamic-concurrent (M ± SD = 0.987 ± 0.648), and static-concurrent (M ± SD = 0.952 ± 0.742). These numerical differences should be interpreted descriptively because the inferential tests were not significant.

#### 3.2.3. Intention Comprehension

The intention comprehension scale showed excellent internal consistency (Cronbach’s α = 0.948) and an adequate KMO value (KMO = 0.769). Presentation form had no significant main effect (F(1, 20) = 0.002, *p* = 0.967, $\eta_{p}^{2}$ < 0.001). Presentation timing had a significant main effect (F(1, 20) = 4.573, *p* = 0.045, $\eta_{p}^{2}$ = 0.186). Intention comprehension was higher under progressive presentation (M ± SD = 5.361 ± 0.818) than under concurrent presentation (M ± SD = 5.107 ± 0.986).

**Figure 18:**
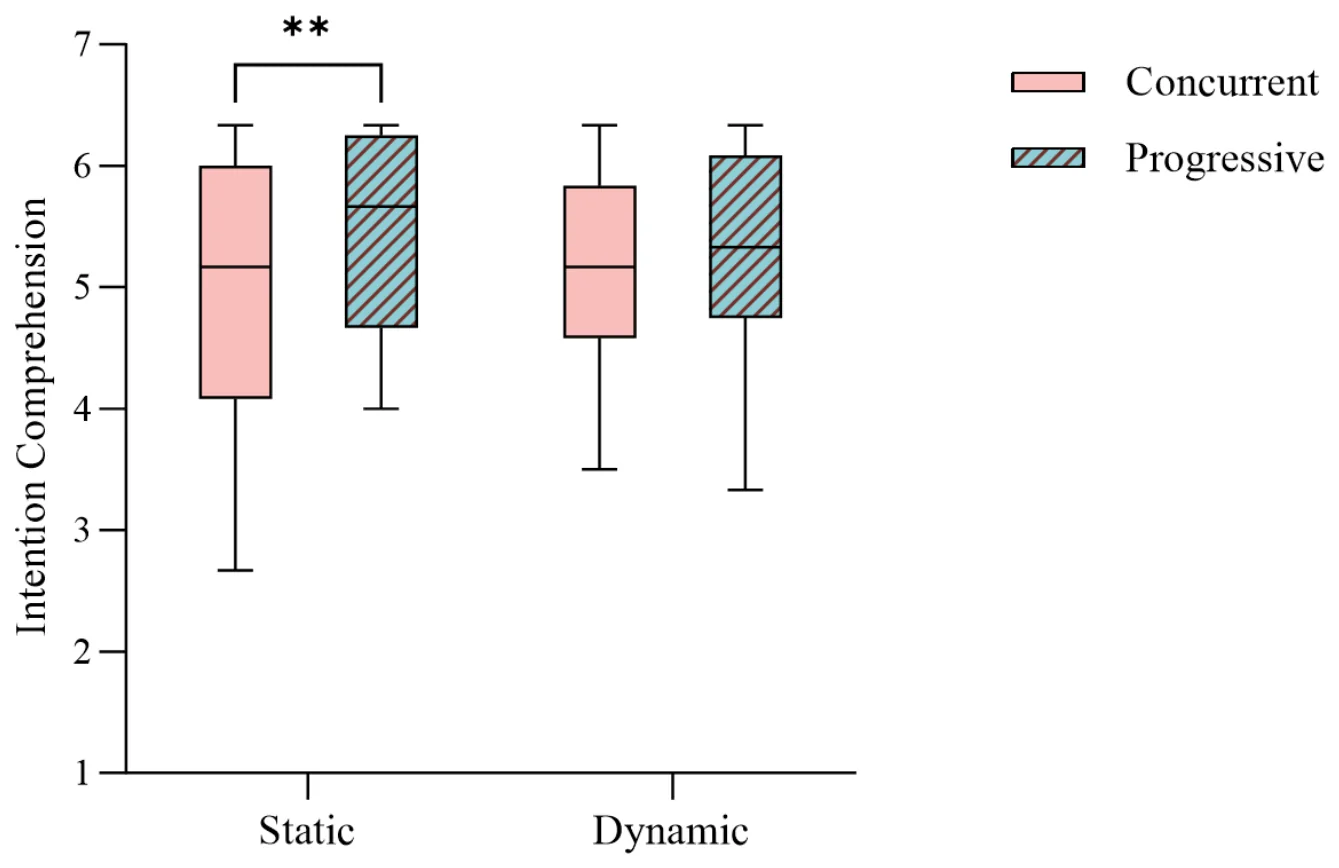
Boxplot of intention comprehension scores under the concurrent and progressive display conditions. (** *p* ≤ 0.01).

The interaction between presentation form and presentation timing was significant (F(1, 20) = 10.000, *p* = 0.005, $\eta_{p}^{2}$ = 0.333). Bonferroni-adjusted simple-effects analyses are shown in Figure 18. Under static presentation, intention comprehension was significantly higher for progressive presentation (M ± SD = 5.468 ± 0.783) than for concurrent presentation (M ± SD = 4.992 ± 1.104, F(1, 20) = 10.127, *p* = 0.005, $\eta_{p}^{2}$ = 0.336). Under dynamic presentation, intention comprehension did not differ significantly between the progressive (M ± SD = 5.254 ± 0.857) and concurrent conditions (M ± SD = 5.222 ± 0.863, F(1, 20) = 0.064, *p* = 0.803, $\eta_{p}^{2}$ = 0.003).

## 4. Discussion

As a spatially overlaid information display technology, AR-HUD may easily induce perceptual competition between virtual elements and the real traffic environment when presenting high-density situational information within a limited takeover time budget. This study combined eye-tracking measures with subjective ratings to examine the effects of presentation form and timing on gaze behavior and interface evaluations. Three main findings emerged. First, progressive presentation produced a more concentrated and orderly gaze pattern and higher perceived usability ratings than concurrent presentation, although several effects depended on presentation form. Second, dynamic presentation did not show a consistent advantage across the measured outcomes, and the longer MFD observed under dynamic presentation may be consistent with greater tracking and information-updating demands. Third, the static-progressive condition showed the most favorable overall descriptive pattern across the measured gaze and rating outcomes, supporting its further evaluation as a candidate presentation configuration.

### 4.1. Effects of Presentation Form on Visual Attention

Presentation form primarily affected MFD. Dynamic presentation produced a significantly longer MFD than static presentation, which may reflect more sustained inspection of continuously changing visual information [40]. One possible interpretation is that dynamic elements may require ongoing visual tracking of spatiotemporal changes and repeated discrimination between virtual icons and the real traffic environment [13,21]. By contrast, the shorter MFD under static presentation may be consistent with more rapid information extraction from stable visual elements and spatial layouts.

Presentation form had no significant main effects on MSA, TTFF, or FC. This pattern may be consistent with a stronger effect of dynamic presentation on sustained visual inspection than on initial visual-search behavior. Although dynamic elements can increase visual salience, dynamic presentation was not associated with shorter TTFF in the present study. Thus, no corresponding evidence of faster initial attentional capture was observed. One possible explanation is that the takeover warning was already highly salient under both presentation forms, leaving limited scope for additional dynamic effects [41].

Presentation form also had no significant main effect on the subjective measures. Thus, the longer fixation durations observed under dynamic presentation were not clearly reflected in participants’ overall interface evaluations. Some drivers may have perceived dynamic elements as useful visual guidance. In addition, retrospective ratings collected after each trial may have been less sensitive to moment-to-moment differences in gaze behavior and may have been influenced by overall impressions and prior expectations [26]. These findings support the combined use of objective eye-tracking measures and subjective evaluations when assessing AR-HUD interfaces.

Dynamic presentation should therefore not be treated as the default format for all takeover information. Static presentation may support more stable gaze allocation by reducing continuous visual changes. Dynamic visual coding may be most appropriate as an initial warning cue [42], whereas stable static presentation may be preferable for subsequent explanations and action guidance.

### 4.2. Effects of Presentation Timing on Visual Information Processing

Presentation timing affected more of the measured gaze and subjective outcomes than presentation form. Compared with concurrent presentation, progressive presentation was associated with lower MFD, MSA, TTFF, and FC. This pattern may reflect more concentrated and sequential gaze allocation, with fewer transitions among information elements. However, the lower FC observed under progressive presentation should be interpreted cautiously. Although the same conceptual takeover-information AOI and nominal 7 s analysis interval were used in all conditions, the amount of visible information and the component-specific exposure durations differed between the two presentation-timing conditions. Thus, the lower FC may reflect both reduced repetitive visual search and reduced information exposure and should not, on its own, be taken as evidence of greater visual-processing efficiency.

Cognitive load theory provides a useful framework for interpreting these findings. Working-memory capacity is limited, and the simultaneous arrival of multiple information units can impose additional selection and integration demands that interfere with subsequent decision-making [43,44]. Concurrent presentation requires drivers to identify, select, and integrate several information elements while repeatedly reallocating limited attentional resources. By dividing the information into consecutive stages, progressive presentation reduced the amount of information visible at any one time. The resulting gaze pattern may be consistent with lower visual-selection and information-integration demands and a more orderly sequence of information acquisition.

The staged release of information may also align with Endsley’s three-level model of situation awareness [3]. During a takeover, drivers must perceive relevant environmental elements, comprehend the current situation, and anticipate future states. Progressive presentation may support this sequence by providing information in an order that corresponds to these processing requirements, reducing the need to integrate several simultaneously presented elements.

The objective and subjective measures showed partial convergence. Progressive presentation reduced all four eye-tracking measures and increased perceived usability and intention comprehension. Interface acceptance, however, did not differ significantly between the presentation timings. Eye-tracking measures may capture moment-to-moment differences in gaze allocation that are not fully reflected in retrospective ratings. Such ratings may be more susceptible to overall impressions, personal preferences, and prior expectations [26]. Consequently, differences in eye-tracking measures may not be accompanied by subjective changes of the same magnitude. The qualitative gaze analysis was also consistent with the quantitative results. Progressive presentation produced a more orderly visual exploration pattern, whereas concurrent presentation was associated with more frequent transitions among information elements.

Together, these findings indicate that progressive presentation promotes more concentrated and sequential gaze allocation and is associated with higher perceived usability ratings. Progressive timing therefore provides a promising candidate strategy for further evaluation in AR-HUD takeover-message design.

### 4.3. Interaction Effects Between Presentation Form and Presentation Timing

Presentation form and presentation timing may be associated with different aspects of gaze behavior during takeover-information processing. The gaze pattern observed under static presentation may be consistent with lower monitoring demands associated with continuous visual changes, whereas progressive presentation reduces the amount of information visible at any one time. These design factors may therefore provide complementary support at different stages of information acquisition.

Significant interactions were observed for MSA, TTFF, and intention comprehension, but not for MFD, FC, perceived usability, or interface acceptance. The combined effects of presentation form and timing therefore appeared to be concentrated in visual search range, initial attentional capture, and intention comprehension. The three significant interactions showed a consistent conditional pattern. The advantages of progressive presentation were primarily observed under static presentation, whereas the corresponding timing differences were not significant under dynamic presentation. Under progressive presentation, static presentation also produced a smaller MSA and shorter TTFF than dynamic presentation. When information is released sequentially, static elements provide stable spatial references. The smaller MSA and shorter TTFF observed under static-progressive presentation may be consistent with more focused gaze on the current target. One possible interpretation is that continuously changing elements prompt repeated visual confirmation and information updating, which may offset some gaze-pattern advantages of progressive presentation. Static presentation and progressive timing were therefore jointly associated with a smaller visual-search range, shorter TTFF, and higher intention-comprehension ratings.

Across the four experimental conditions, the static-progressive condition showed the most favorable overall descriptive pattern in the eye-tracking and subjective measures. The combination of stable visual presentation and staged information delivery was associated with more focused gaze allocation and favorable perceived usability and intention comprehension ratings. This pattern supports prioritizing the static-progressive condition for further prototype evaluation.

Compared with recent studies of multimodal takeover requests, the present findings emphasize the importance of information organization within the visual modality. Previous research has primarily examined the selection and combination of visual, auditory, and tactile channels [15,16,17]. The present study indicates that presentation form and timing can also jointly shape gaze allocation and subjective evaluations within a single visual channel. Takeover-interface design should therefore consider not only how sensory modalities are combined but also how information is organized within each modality.

Based on the observed gaze and rating patterns, presentation form and timing warrant joint consideration in subsequent AR-HUD prototype evaluation. Static-progressive presentation could serve as a candidate for subsequent prototype development and on-road validation. Key information could be released sequentially according to a “warning–cause explanation–action guidance” structure, thereby reducing competition among simultaneously displayed elements. Dynamic elements may be useful as salient cues for initial warnings, but their continuous use may be associated with more sustained visual inspection. Future AR-HUD interfaces should therefore balance visual salience against the potential need for sustained inspection, while ensuring that risk information is communicated promptly.

### 4.4. Implications for Adaptive AR-HUD Design

From a practical design perspective, original equipment manufacturers (OEMs) and HMI designers may consider static-progressive presentation as a candidate configuration for prototype development. In such prototypes, takeover information could be released sequentially according to a “warning–cause explanation–action guidance” structure. Dynamic elements may be selectively evaluated when rapid attentional capture or immediate risk communication is required. However, validation in high-fidelity simulations and actual takeover tasks is required before static-progressive presentation can be recommended as a default strategy for production AR-HUD systems.

The present findings also have implications for next-generation AR-HUD interface design in conditional automated driving. The observed trade-offs between static and dynamic presentation suggest that a fixed presentation strategy may not be optimal across all driving contexts. Instead, interface design may need to account for differences in information requirements and situational demands. This consideration supports the development of real-time, state-aware adaptive AR-HUD systems that tailor information presentation to drivers’ momentary needs [45].

Building upon these findings, a promising direction is the development of a closed-loop human-in-the-loop framework that continuously monitors driver states and adaptively regulates interface behavior. Such a framework could integrate multimodal sensing technologies, including non-intrusive eye-tracking metrics (e.g., fixation stability and pupil diameter), physiological signals (e.g., HRV and EEG), and vehicle control data, to estimate drivers’ cognitive workload, visual attention, and situation awareness in real time [45,46]. Based on these estimates and the urgency of the driving context, adaptive decision logic could dynamically select the most appropriate presentation strategy.

### 4.5. Limitations and Future Work

This study has several limitations. First, the experiment used prerecorded video stimuli in a controlled laboratory environment. This approach provided experimental control and supported stable eye-tracking measurements. However, unlike real automated driving takeovers, participants were not required to control a vehicle and were not exposed to the consequences, perceived risk, or psychological pressure associated with actual traffic hazards. The study also used a 7 s takeover time budget (TTB) to represent a relatively urgent takeover situation. Although this setting allowed for gaze behavior and subjective evaluations to be examined under time pressure, real takeover events vary in urgency, duration, and complexity. Future studies should examine different TTBs and more complex driving scenarios using high-fidelity simulators, closed-road tests, or naturalistic driving studies.

Second, the amount and duration of information visible within the overall takeover-information AOI differed between the two presentation-timing conditions. Although the same conceptual takeover-information AOI and 7 s analysis interval were used in all conditions, all three information components were visible from TOR onset under concurrent presentation, whereas the Why and How components appeared later under progressive presentation. Consequently, the lower full-trial FC observed under progressive presentation may partly reflect the smaller amount of information available for inspection rather than greater visual-processing efficiency. Because component-specific eye-tracking measures were not calculated, the present analysis cannot fully distinguish between these explanations. Future studies should employ component-specific AOIs, onset-aligned observation windows, or appropriately exposure-normalized measures to separate the effects of presentation timing from those of information exposure.

Third, the sample was relatively small and consisted mainly of drivers aged 20–30 years. The findings may therefore not generalize to the broader driving population. Older drivers may differ in visual ability, processing speed, and attentional-resource allocation, resulting in different information-acquisition patterns during automated driving takeovers. Previous research on augmented-reality interaction for older users indicates that larger visual elements, simpler interface hierarchies, less visual clutter, and multimodal cues may improve readability and usability [47,48]. These findings may also inform age-inclusive AR-HUD design.

All participants were recruited from the Chinese driving population. The results may therefore have been influenced by local traffic culture, driving habits, and familiarity with automated driving systems. Future research should include larger and more diverse samples spanning age groups, driving experience, and familiarity with advanced driver-assistance systems. Cross-cultural and cross-environmental studies are also needed to examine the generalizability of the findings.

Fourth, this study focused on visual presentation strategies, whereas real takeover requests often combine visual, auditory, and tactile information [49]. Future research should examine interactions among these modalities and determine how information can be distributed across sensory channels without creating redundant or conflicting demands.

Finally, the study used a single-session design and primarily examined participants’ initial exposure to the AR-HUD takeover interface. It therefore could not capture learning and adaptation during extended use. As drivers become familiar with the information structure, prompting logic, and visual feedback, their search strategies, comprehension, and acceptance may change. Longitudinal studies with repeated measurements are needed to evaluate the effectiveness and stability of the presentation strategies over time.

## 5. Conclusions

This study combined eye tracking with subjective rating scales to systematically examine how presentation form (static vs. dynamic) and presentation timing (concurrent vs. progressive) affected gaze behavior and subjective evaluations while 21 licensed drivers viewed prerecorded AR-HUD takeover scenarios. Compared with concurrent presentation, progressive presentation produced lower MFD, MSA, TTFF, and FC and higher perceived-usability ratings, a pattern that may reflect more concentrated and orderly gaze allocation. The lower FC may partly reflect the reduced amount of information available for inspection during part of the progressive condition. Dynamic presentation was not associated with shorter TTFF and produced a longer MFD than static presentation, which may reflect more sustained visual inspection. Among the four experimental conditions, the static-progressive condition showed the most favorable overall descriptive profile across the eye-tracking and subjective measures. Overall, presentation timing shaped a broader range of gaze and subjective outcomes than presentation form. The observed patterns support further evaluation of progressive information organization, particularly when combined with stable static presentation, as a candidate strategy for AR-HUD takeover-message design. Future high-fidelity simulations and active takeover tasks can extend these findings to workload, driving performance, and safety outcomes.

## Figures and Tables

**Table 1:** Subjective evaluation measures for takeover interface presentation strategies.

Measure	Questionnaire Item	Reference
PU	The information presented by the interface is clear.	[37]
	The information presented by the interface is easy to read.	
	The information presented by the interface is easy to understand.	
	The information presented by the interface is well integrated.	
	The interface presents too much information.	
VDLAS	Useful—Useless	[38]
	Pleasant—Unpleasant	
	Good—Bad	
	Enjoyable—Annoying	
	Effective—Ineffective	
	Likeable—Unlikeable	
	Supportive—Worthless	
	Acceptable—Unacceptable	
	Increases alertness—Induces drowsiness	
IC	The interface clearly conveys the vehicle’s intended actions.	[39]
	The order of information presentation matches my cognitive process.	
	The vehicle’s behavior is consistent with the information presented by the interface.	

## Data Availability

The original contributions presented in this study are included in the article/Supplementary Material. Further inquiries can be directed to the corresponding author.

## Supplementary Materials

The following supporting information can be downloaded at https://www.mdpi.com/article/10.3390/jemr19040092/s1, Figure S1: Takeover-information AOI overlays and coordinate definitions for the eight experimental conditions; Table S1: Eye-tracking data quality at the participant, trial, and experimental-condition levels; Video S1–S8: Stimulus videos covering the static-concurrent, static-progressive, dynamic-concurrent, and dynamic-progressive conditions in the vehicle breakdown and road construction scenarios.

## Abbreviations

The following abbreviations are used in this manuscript:

AR-HUD	Augmented Reality Head-Up Display
HMI	Human–Machine Interface
L3	Level 3
ADAS	Advanced Driver Assistance System
TOR	Takeover Request
TTB	Takeover Time Budget
NDRT	Non-Driving-Related Task
SAT	Situation Awareness-Based Agent Transparency
AOI	Area of Interest
MFD	Mean Fixation Duration
MSA	Mean Saccade Amplitude
TTFF	Time to First Fixation
FC	Fixation Count
I-VT	Identification by Velocity Threshold
