# Supplementary material for: Effects of Presentation Form and Presentation Timing in AR-HUD Takeover Displays on Driver Visual Attention: An Eye-Tracking Study
Source: J Eye Mov Res. 2026 Aug 20;19(4):92. doi: 10.3390/jemr19040092 (PMC13513926; doi:10.3390/jemr19040092)
Supplement: Supplementary file 1 [file jemr-19-00092-s001.zip › Figure S1.pdf]

Supplementary Figure S1 - Test 1: Vehicle breakdown / SC

Red rectangle: active overall takeover-information AOI. Coordinate origin is the top-left of the 1920 x 1080 frame.
The trajectory line is displayed as part of the stimulus but is not included in the quantitative AOI.

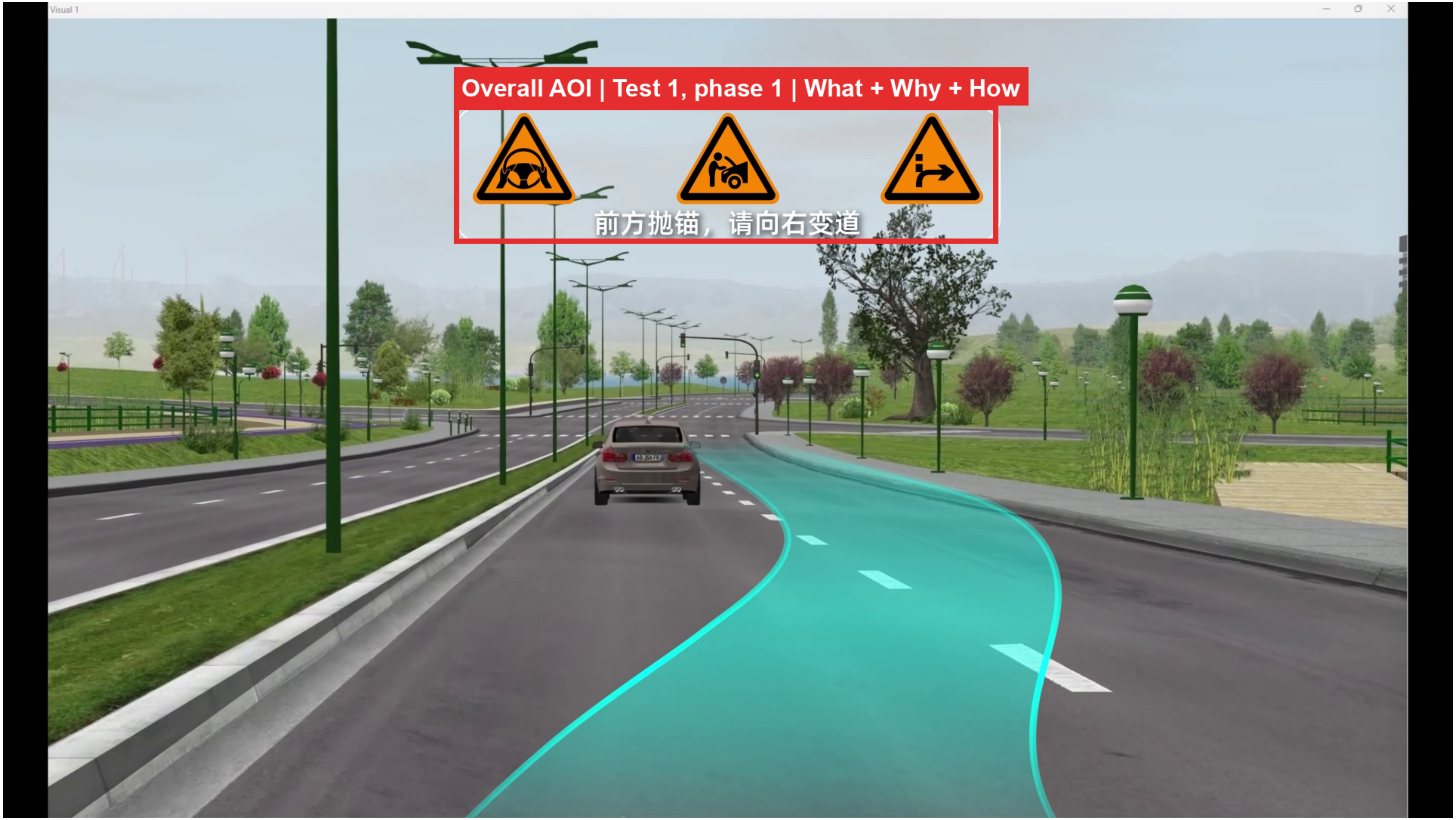

Phase 1: What + Why + How | nominal 0.0-7.0 s

x=597.194, y=136.671, w=720.495, h=182.724 px

stored media time 1.750-8.684 s

Supplementary Figure S1 - Test 2: Vehicle breakdown / DC

Red rectangle: active overall takeover-information AOI. Coordinate origin is the top-left of the 1920 x 1080 frame.
The trajectory line is displayed as part of the stimulus but is not included in the quantitative AOI.

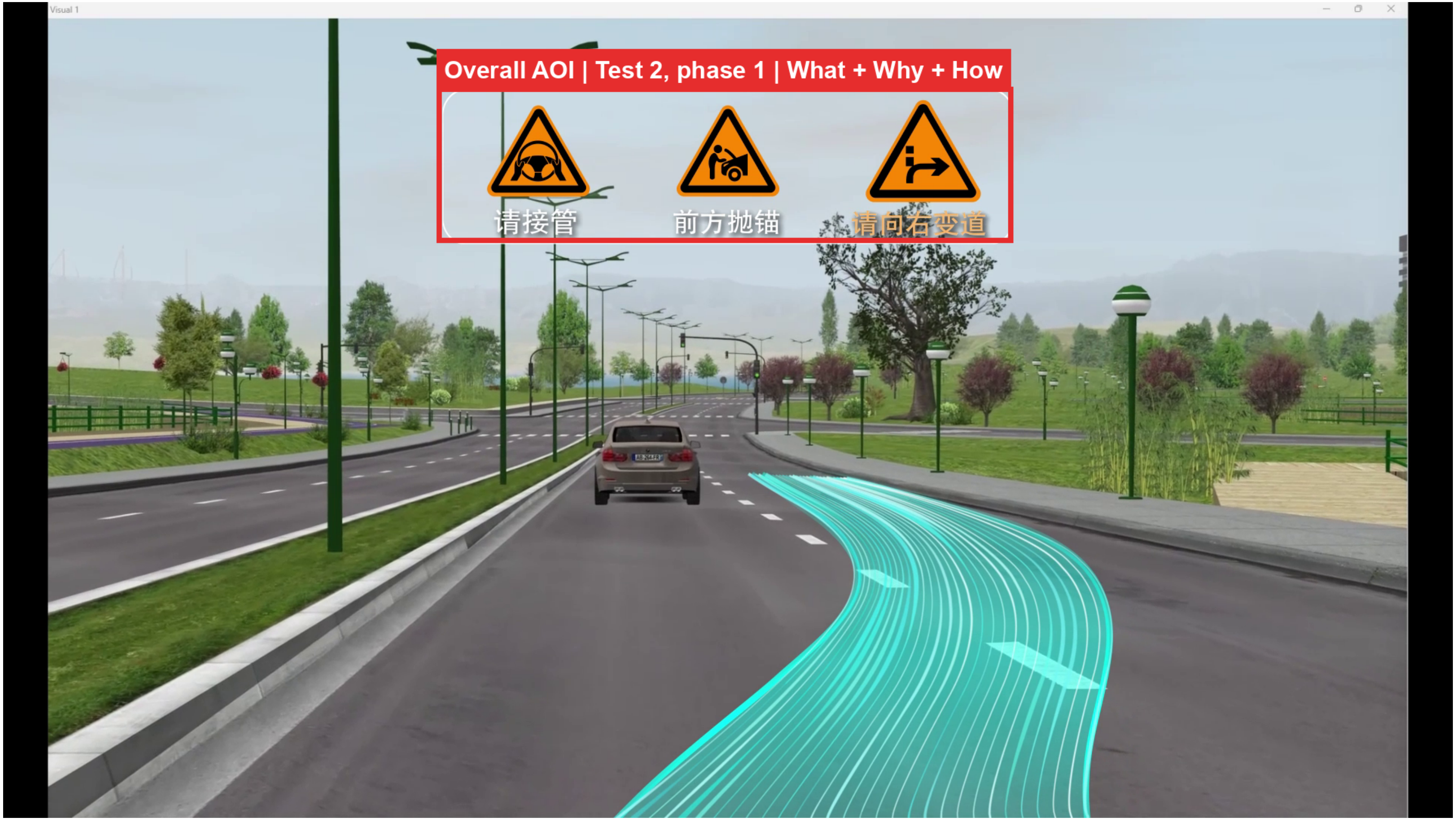

Phase 1: What + Why + How | nominal 0.0-7.0 s

x=574.916, y=112.902, w=762.085, h=205.896 px

stored media time 1.773-8.833 s

Supplementary Figure S1 - Test 3: Vehicle breakdown / DP

Red rectangle: active overall takeover-information AOI. Coordinate origin is the top-left of the 1920 x 1080 frame.
The trajectory line is displayed as part of the stimulus but is not included in the quantitative AOI.

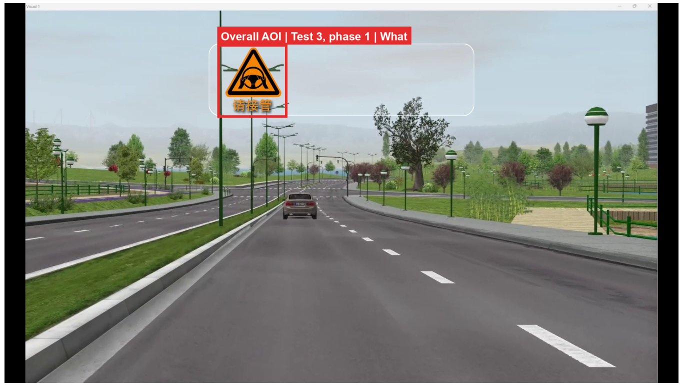

Phase 1: What | nominal 0.0-1.5 s

x=606.107, y=117.359, w=199.065, h=207.978 px
stored media time 1.772-3.318 s

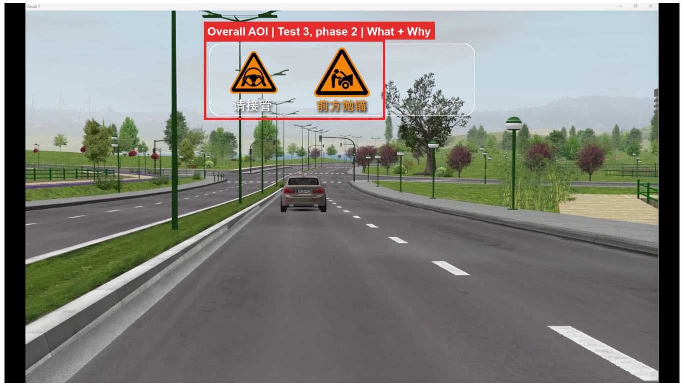

Phase 2: What + Why | nominal 1.5-3.0 s

x=567.483, y=103.989, w=515.488, h=228.776 px
stored media time 3.318-4.846 s

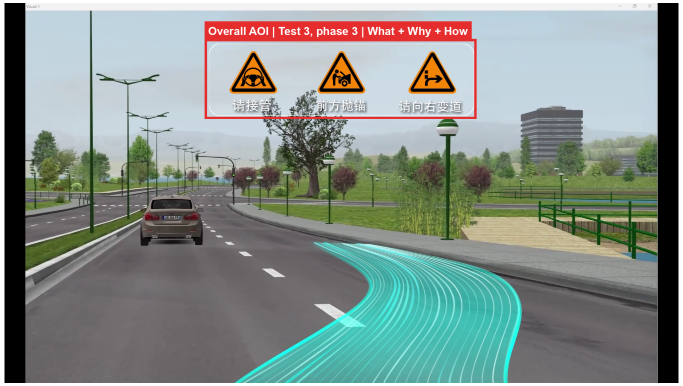

Phase 3: What + Why + How | nominal 3.0-7.0 s

x=570.454, y=102.503, w=773.975, h=227.290 px
stored media time 4.846-8.824 s

Supplementary Figure S1 - Test 4: Vehicle breakdown / SP

Red rectangle: active overall takeover-information AOI. Coordinate origin is the top-left of the 1920 x 1080 frame.
The trajectory line is displayed as part of the stimulus but is not included in the quantitative AOI.

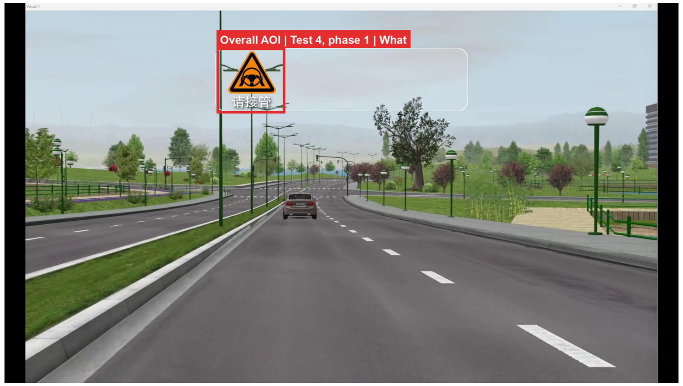

Phase 1: What | nominal 0.0-1.5 s

x=604.622, y=127.758, w=194.608, h=185.695 px
stored media time 1.772-3.305 s

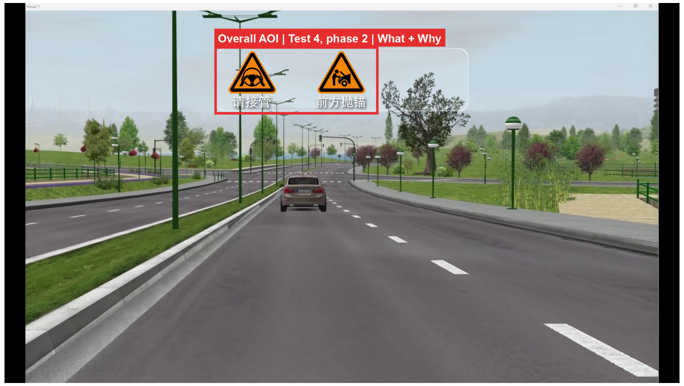

Phase 2: What + Why | nominal 1.5-3.0 s

x=597.194, y=123.301, w=466.465, h=193.122 px
stored media time 3.305-4.805 s

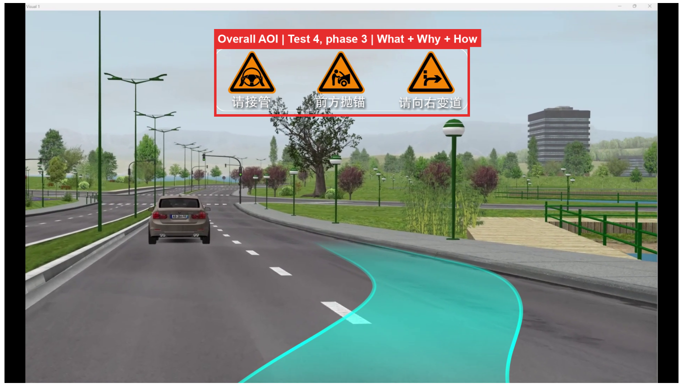

Phase 3: What + Why + How | nominal 3.0-7.0 s

x=597.194, y=124.787, w=727.923, h=197.579 px
stored media time 4.805-8.779 s

Supplementary Figure S1 - Test 5: Road construction / SC

Red rectangle: active overall takeover-information AOI. Coordinate origin is the top-left of the 1920 x 1080 frame.
The trajectory line is displayed as part of the stimulus but is not included in the quantitative AOI.

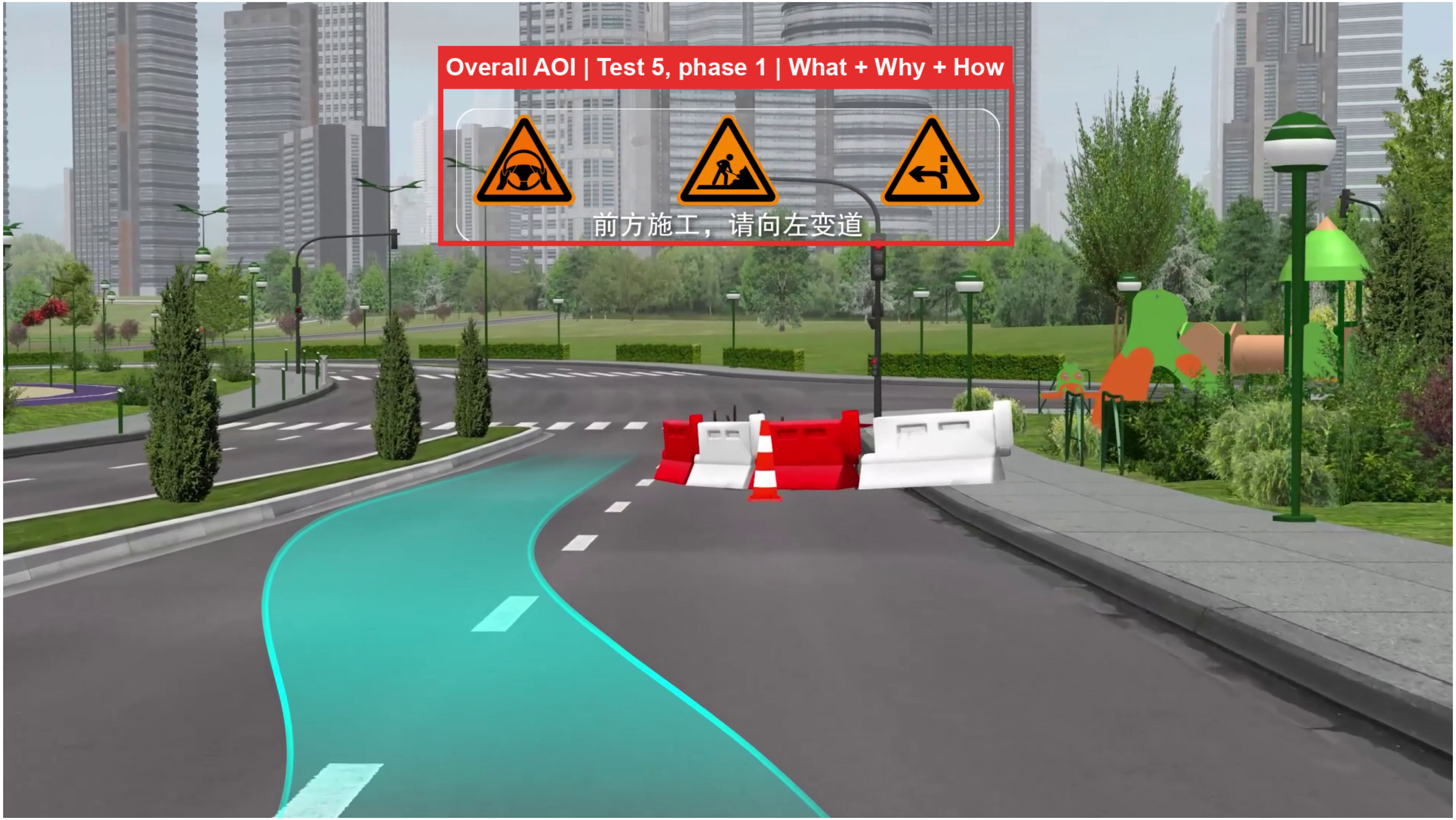

Phase 1: What + Why + How | nominal 0.0-7.0 s

x=576.396, y=108.446, w=762.091, h=213.920 px

stored media time 3.211-10.193 s

Supplementary Figure S1 - Test 6: Road construction / DP

Red rectangle: active overall takeover-information AOI. Coordinate origin is the top-left of the 1920 x 1080 frame.
The trajectory line is displayed as part of the stimulus but is not included in the quantitative AOI.

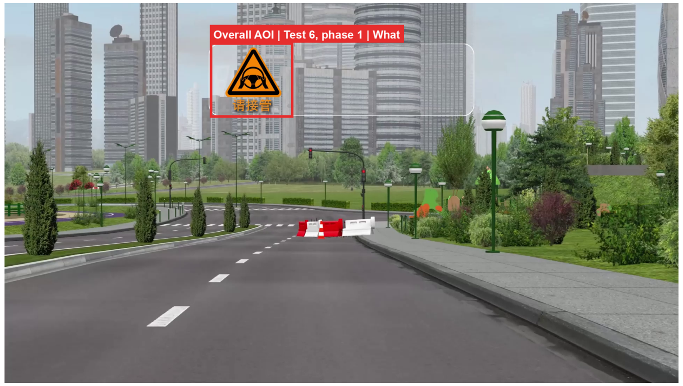

Phase 1: What | nominal 0.0-1.5 s

x=585.310, y=112.902, w=237.689, h=212.435 px

stored media time 3.207-4.744 s

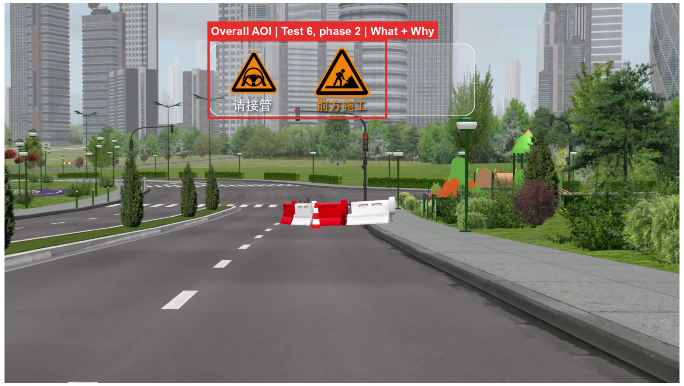

Phase 2: What + Why | nominal 1.5-3.0 s

x=577.882, y=102.503, w=511.032, h=227.290 px

stored media time 4.744-6.277 s

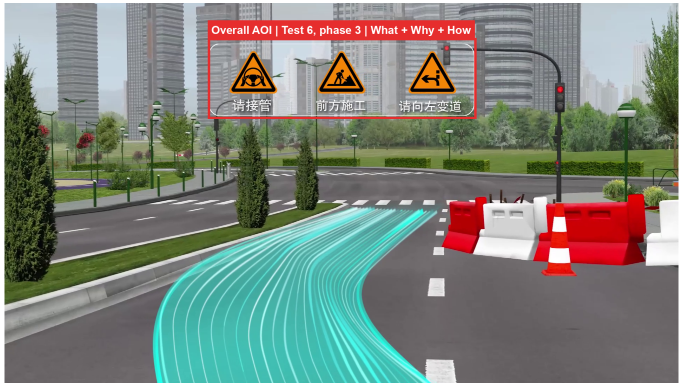

Phase 3: What + Why + How | nominal 3.0-7.0 s

x=579.367, y=99.532, w=765.062, h=230.261 px

stored media time 6.277-10.236 s

Supplementary Figure S1 - Test 7: Road construction / DC

Red rectangle: active overall takeover-information AOI. Coordinate origin is the top-left of the 1920 x 1080 frame.
The trajectory line is displayed as part of the stimulus but is not included in the quantitative AOI.

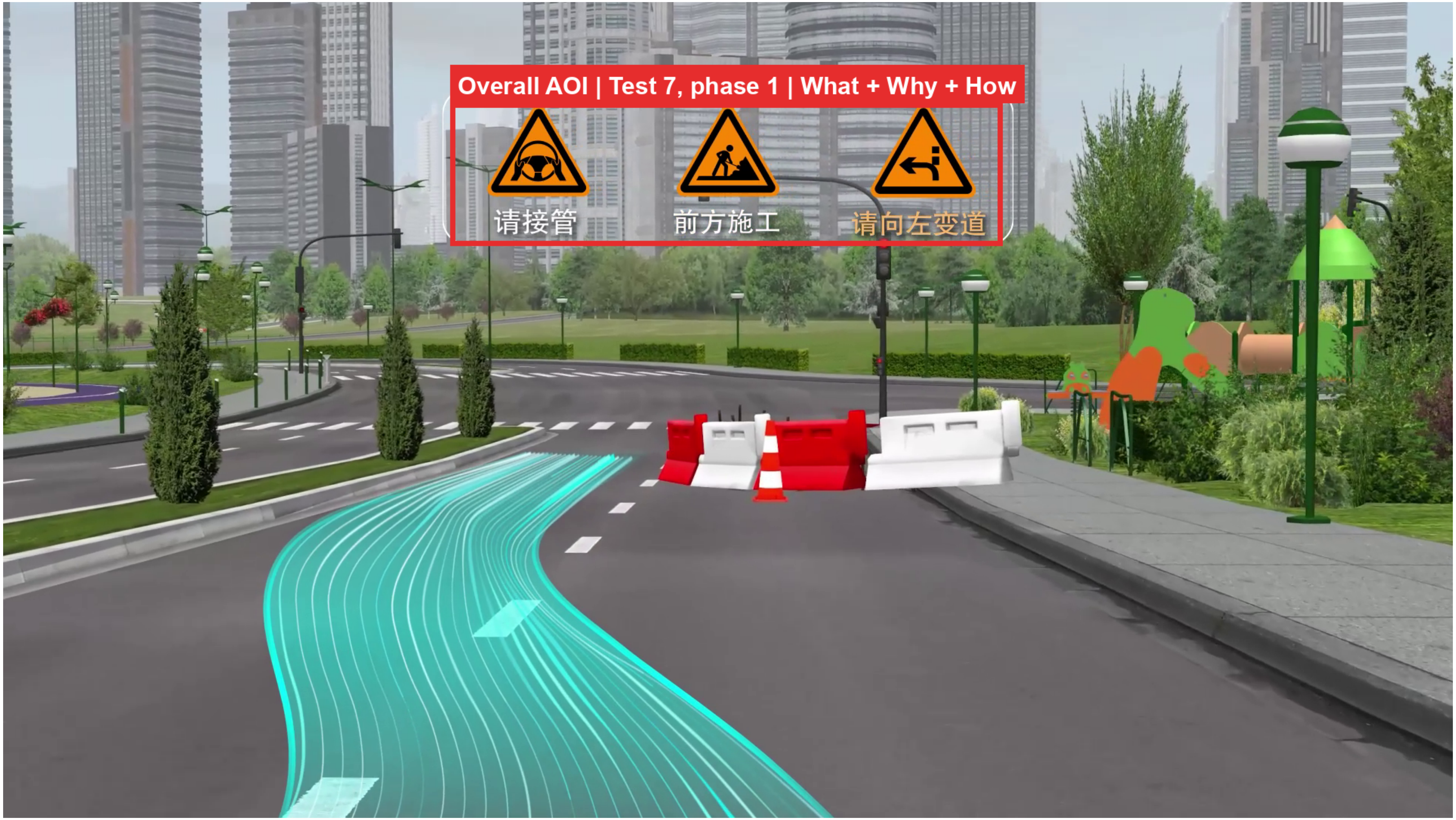

Phase 1: What + Why + How | nominal 0.0-7.0 s

x=592.737, y=133.700, w=730.894, h=188.666 px

stored media time 3.205-10.234 s

Supplementary Figure S1 - Test 8: Road construction / SP

Red rectangle: active overall takeover-information AOI. Coordinate origin is the top-left of the 1920 x 1080 frame.
The trajectory line is displayed as part of the stimulus but is not included in the quantitative AOI.

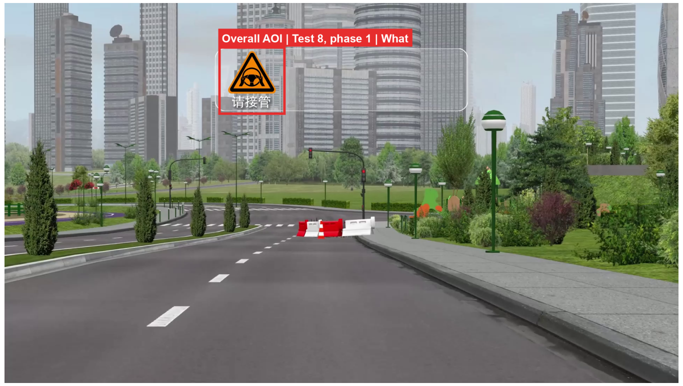

Phase 1: What | nominal 0.0-1.5 s

x=609.078, y=123.301, w=191.637, h=194.608 px
stored media time 3.216-4.742 s

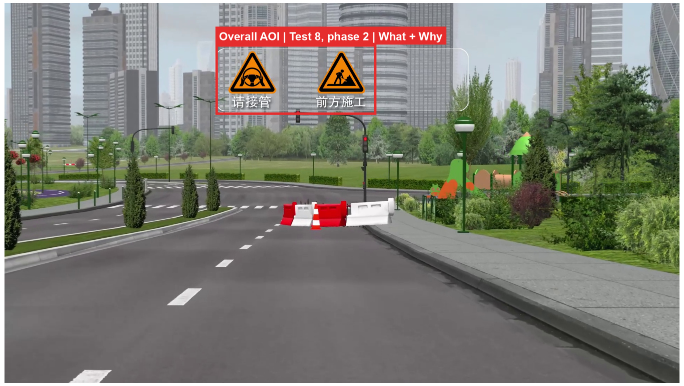

Phase 2: What + Why | nominal 1.5-3.0 s

x=600.165, y=117.359, w=457.552, h=200.550 px
stored media time 4.742-6.247 s

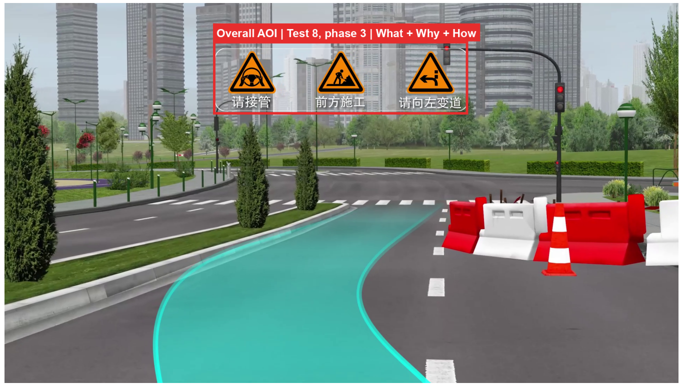

Phase 3: What + Why + How | nominal 3.0-7.0 s

x=594.223, y=108.446, w=726.437, h=207.978 px
stored media time 6.247-10.236 s
